# Overall *in vitro, in vivo*, and *in silico* evaluation of *Olea europaea* and *Ficus carica* leaf extracts for antimicrobial activity against multidrug-resistant pathogens

**DOI:** 10.3389/fmicb.2025.1567921

**Published:** 2025-05-19

**Authors:** Mahmoud Aloriby, Mohamed Elkawafi, Salem Aldrsy, Mohamed Sweker, Hadeel Elabdeli, Aisha Elbarghathi, Ahmed Benhasouna, Madiha El-Awamie, Nariman Elsharif, Omar Alqabbasi, Salmin Alshalmani, Rabiea Algazal, Farag Bleiblo

**Affiliations:** ^1^Department of Cytotechnology, Faculty of Biomedical Sciences, University of Benghazi, Benghazi, Libya; ^2^Department of Pathology, Medical Center, Libyan International Medical University, Benghazi, Libya; ^3^Basic Medical Sciences Program, Faculty of Medical and Health Sciences, Libyan International Medical University, Benghazi, Libya; ^4^Department of Pathology, Faculty of Medicine, University of Benghazi, Benghazi, Libya; ^5^Department of Microbiology, Faculty of Science, University of Benghazi, Benghazi, Libya; ^6^Department of Molecular Diagnostics, Faculty of Biomedical Sciences, University of Benghazi, Benghazi, Libya; ^7^Department of Pharmacognosy, Faculty of Pharmacy, University of Benghazi, Benghazi, Libya; ^8^Laboratory of Immunology and Virology, Children Hospital of Benghazi, Benghazi, Libya

**Keywords:** *Olea europae*, *Ficus carica*, multidrug-resistant pathogens, solvent extraction, antimicrobial activities, toxicological evaluation, histopathology, *in silico* modeling and docking

## Abstract

**Introduction:**

Antimicrobial resistance (AMR) represents a critical global health issue, prompting the urgent exploration of alternative plant-derived antimicrobial therapies. In this context, the present study evaluates the therapeutic efficacy and safety profiles of *Olea europaea* and *Ficus carica* leaf extracts against multidrug-resistant pathogens, integrating *in vitro* antimicrobial assays, *in vivo* toxicity assessments, and *in silico* modeling approaches.

**Methods:**

Leaf extracts from *O. europaea* and *F. carica* were prepared by solvent-based maceration using methanol, acetone, and distilled water. Their antimicrobial properties were evaluated through disk and well diffusion assays to determine the minimum inhibitory concentration (MIC) and minimum bactericidal concentration (MBC) against clinically relevant pathogens. Toxicological assessments were performed *in vivo* using the BALB/c mice model, including histopathological examinations, hematological profiling, and biochemical analyses. A complementary *in vitro* toxicogenomic screening was conducted using a cell-based reporter assay to profile nuclear receptor signaling and cellular stress responses. Furthermore, computational modeling and molecular docking were employed to predict the possible interactions of selected phytochemicals with *E. coli* cytochrome c peroxidase.

**Results:**

Methanolic extracts of *O. europaea* exhibited potent antimicrobial activity against multidrug-resistant isolates, whereas *F. carica* extracts showed minimal efficacy across all experimental contexts. *In silico* molecular docking analyses revealed high-affinity interactions between olive-derived phenolic compounds and *E. coli* cytochrome c peroxidase, suggesting a plausible mechanistic basis for the observed antibacterial effects. *In vivo*, toxicological evaluation in BALB/c mice administered aqueous formulations of the methanolic olive extract demonstrated dose-dependent hepatic and renal histopathological alterations, accompanied by dysregulation of the immunological profiles and elevated hepatic enzyme levels. These findings were consistent with outcomes from the cell reporter assays and computational toxicology models, which indicated potential nephrotoxic and immunotoxic risks at higher concentrations.

**Discussion:**

These findings validate the promising antimicrobial activity of *O. europaea* and *F. carica* leaf extracts against multidrug-resistant pathogens. However, further investigations on precise dosage optimization and long-term safety evaluations are essential before these extracts are implemented in clinical practice.

## 1 Introduction

Antimicrobial resistance (AMR) has become a leading cause of mortality globally, with an estimated 1.14 million deaths directly caused by drug-resistant bacterial infections in 2021. In the absence of effective interventions, this figure is anticipated to rise significantly, potentially resulting in ~2 million direct deaths annually by 2050 (Sullivan et al., 2024). Therefore, an integrated strategy emphasizing improved antimicrobial stewardship, infection control measures, and innovative therapeutic approaches is essential to effectively address this challenge (Ho et al., 2024). Recent reports highlight exploring alternative antimicrobial strategies, including plant-based pharmaceuticals, bacteriophage therapy, antimicrobial peptides, RNA-based therapies, and probiotics (Singha et al., 2024). Several plant-derived phytochemicals have shown promising activity against multidrug-resistant (MDR) pathogens as direct inhibitors or by potentiating the action of traditional antimicrobial drugs to overcome resistance mechanisms (Jubair et al., 2021). The renewed interest in plant-derived antimicrobials reflects a strategic paradigm shift in antimicrobial drug discovery, emphasizing their potential as rich reservoirs of novel, bioactive compounds to address the rising challenge of MDR. The secondary metabolites of plants offer considerable therapeutic promise due to their inherent biocompatibility.

Olive (*O. europaea*) leaf extracts exhibit broad-spectrum antibacterial properties, demonstrating significant *in vitro* efficacy against pathogens commonly associated with gastrointestinal and respiratory tract infections (de Oliveira et al., 2024). The antimicrobial efficacy of olive leaf extract is attributed to a synergistic interplay of its phenolic constituents (like oleuropein and hydroxytyrosol) and other components, such as fatty acids (de Oliveira et al., 2024). Recent studies highlight the potent antimicrobial activity of olive leaf extract, revealing an 82% inhibition of biofilm formation by MDR *Pseudomonas aeruginosa* strains at sub-inhibitory concentrations (Esfandiary et al., 2024). Furthermore, olive leaf extracts have demonstrated potent bactericidal activity against *Campylobacter* species resistant to ciprofloxacin and tetracycline (Silvan et al., 2022). Similarly, fig (*F. carica*) leaves are a potential source of diverse bioactive compounds with promising antimicrobial and therapeutic activities. Phytochemical analyses reveal that fig leaves contain various organic acids, coumarins, tannins, and flavonoids, contributing to their ethnomedicinal uses (Shiraishi et al., 2023). Notably, *F. carica* leaf extracts exhibited potent antimicrobial activity against several MDR pathogens, including carbapenem-resistant *Klebsiella pneumoniae, Escherichia coli, Staphylococcus aureus*, and *P. aeruginosa* (Shiraishi et al., 2023). The fig extract was particularly effective due to the phenolic compounds, including eugenol, acetyleugenol, and psoralen, which were demonstrated to be the essential active constituents (Kim and Lee, 2023). Collectively, *F. carica* leaf extracts demonstrate antimicrobial efficacy and pharmacological activity, underscoring their potential utility as alternative therapies in managing infections caused by MDR pathogens.

This investigation employs a comprehensive strategy to integrate *in silico* modeling, *in vitro* experiments, and *in vivo* validation to assess the antimicrobial potential and safety of *O. europaea* and *F. carica* leaf extracts. Computational techniques enable efficient prediction of interactions between phytochemicals and biological targets (Mangana et al., 2025; Mishra and Muthukaliannan, 2024), while *in vitro* analyses provide empirical evidence of efficacy against MDR organisms (Esfandiary et al., 2024). Subsequent *in vivo* studies in animal models provide critical insights into therapeutic efficacy and biosafety, enhancing the translational relevance of the findings (Shiraishi et al., 2023). Although *in silico, in vitro*, and *in vivo* approaches have been independently explored in previous studies, research integrating these methodologies remains limited. This study conducts a comprehensive evaluation to underscore the therapeutic potential of *O. europaea* and *F. carica* leaf extracts as plant-based interventions against antimicrobial resistance.

## 2 Materials and methods

### 2.1 *In vitro* studies

#### 2.1.1 Antimicrobial susceptibility testing

Antimicrobial susceptibility profiles for selected clinically relevant pathogens were evaluated against a panel of antimicrobial agents. Antimicrobial susceptibility testing was performed using the standardized disk diffusion method according to the Clinical and Laboratory Standards Institute (CLSI) guidelines (Haley et al., 2024). Bacterial and fungal isolates from clinical samples were identified using standard methods. The pathogens investigated in the study were *Escherichia coli, Klebsiella pneumoniae, Staphylococcus aureus, Enterococcus faecalis, Enterococcus faecium, Streptococcus agalactiae, Enterobacter cloacae*, and *Candida albicans*. The clinical isolates were first cultivated on appropriate selective and enriched media for optimal growth and preliminary identification. Subsequent identification of the pathogens was conducted using the Vitek2 Compact Identification System (BioMérieux, Marcy-l'Étoile, France) for confirmation. The selected antimicrobial agents included azithromycin (AZM), nitrofurantoin (NIF), trimethoprim/sulfamethoxazole (SXT), amikacin (AK), gentamicin (CN), vancomycin (VA), amoxicillin/clavulanic acid (AMC), ceftriaxone (CRO), ceftazidime (CAZ), and tobramycin (TOP; Oxoid Ltd., Basingstoke, UK). These antimicrobial agent were chosen based on their clinical relevance and therapeutic significance. Inoculum suspensions (in 0.5 McFarland unit) were plated on Mueller-Hinton agar (for bacteria) and Sabouraud Dextrose Agar (SDA) for fungi to perform the test (Kebede and Shibeshi, 2022). The inhibition zone diameters were measured in millimeters and interpreted as Susceptible (S) and Resistant (R) according to CLSI guidelines.

#### 2.1.2 Plant material and extraction procedures

##### 2.1.2.1 Sample collection of plant material

*O. europaea* leaves were collected in July 2024 from a 12-year-old Manzanilla del Litoral tree in the Abuatni district of Benghazi, Libya. Simultaneously, *F. carica* leaves were collected from a 28-year-old Moraceae tree in the Al-Laithi district of the same city. Before analysis, all plant materials were thoroughly washed with running tap water to remove superficial debris and then air-dried at ambient temperature in a shaded and well-ventilated area for 6 days. The leaves were dried in a dehydrator before being ground to a fine powder in a commercial blender (Ahmed et al., 2023).

##### 2.1.2.2 Extraction protocol

For each plant species, 20 g of powdered olive leaves and 15 g of powdered fig leaves were accurately weighed and individually immersed in 200 mL of high-purity analytical-grade methanol or acetone (BDH Chemicals Ltd., England) or distilled water. The extraction was performed in sterile glass containers under controlled laboratory conditions to ensure reproducibility and prevent contamination. We selected methanol, acetone, and distilled water based on their efficacy in extracting polyphenolic compounds from plant materials, as supported by previous studies. The solvent-to-powder ratio was optimized following standard extraction protocols to ensure efficient recovery of bioactive constituents and allow methodological consistency across conditions. These parameters are widely used in phytochemical extraction to maximize yield and reproducibility (Cifá et al., 2018; Meziant et al., 2018; Agatonovic-Kustrin et al., 2023; Nguyen et al., 2023). The mixtures were kept at room temperature for 24 h, with occasional gentle agitation, to facilitate the extraction of bioactive compounds. After maceration, the extracts were first filtered through multiple layers of gauze and subsequently passed through 180 mL of filter paper to remove coarse particulates.

##### 2.1.2.3 Concentration and stock solution preparation

The resulting filtrates were concentrated under reduced pressure with a rotary evaporator at 45°C for methanolic and acetone extracts and at 100°C for the distilled water extract. The acetone and methanolic residues were left at room temperature for 24 h without covering to allow residual solvents to evaporate. Meanwhile, the distilled water extract was further concentrated via Soxhlet extraction until a sufficiently dense residue was obtained. For the olive leaf extract, an approximate yield of 4 g was achieved from the initial leaf powder at a solvent-to-powder ratio of 1:5. A total of 2 g of fig leaf extract was obtained at a solvent-to-powder ratio of 1:7.5 following the extraction process, providing an adequate quantity for subsequent antimicrobial evaluation and toxicity assessment. The lower yield of *F. carica* extract may be attributed to the comparatively lower concentration of extractable bioactive compounds, mainly phenolic acids, and flavonoids, which vary depending on the plant's maturity, growing conditions, and solvent polarity. Fig leaves also possess a different cellular structure than olive leaves, which may affect solvent permeability and compound solubility (Cho et al., 2020; Khelouf et al., 2023; Zhang et al., 2024). Notably, during our preliminary experiments using different solvents under the same conditions, we did not observe significant variations in extraction yields, suggesting that the solvent type may not significantly influence the yield differences between the two plant species (Abi-Khattar et al., 2019). The dried fine powder from each extract was reconstituted in dimethyl sulfoxide (DMSO) to prepare a stock solution at 100 mg/mL concentration and transferred into dark, airtight vials for storage at room temperature until further use.

##### 2.1.2.4 Disc and well diffusion assay

Sterile filter-paper discs were prepared by punching uniform circles (6 mm diameter) and immersing them in each extract solution for 12 h. Standardized Muller-Hinton agar plates were bored with 6 mm wells and filled with the corresponding volumes or concentrations of olive or fig leaf extract for well-diffusion assays. All prepared plates were subsequently sealed and stored at 4°C in sterile amber glass vials, protected from light and moisture until antimicrobial testing commenced within 7–10 days of preparation to ensure chemical stability and preserve bioactivity (Sa and Bradford, 2008; Ahmad-Qasem et al., 2016; Khelouf et al., 2023).

#### 2.1.3 Cultivation and standardization of microbial strains

##### 2.1.3.1 Bacterial cultures

The clinical samples used in this study were collected from hospitalized patients with confirmed bacterial infections. Isolates of Gram-positive and Gram-negative bacteria were collected from various clinical specimens from patients of different ages and sexes, including stool, blood, cerebrospinal fluid (CSF), urine, and swab cultures. All isolates were obtained with informed patient consent and approved by the institutional ethics committee to ensure compliance with ethical research standards. Bacterial identification was performed using the Vitek2 compact system (BioMérieux, Marcy-l'Étoile, France). The Gram-positive isolates included *Staphylococcus aureus, Streptococcus agalactiae*, and *Enterococcus faecalis*, while the Gram-negative isolates comprised *Escherichia coli, Pseudomonas aeruginosa*, and *Klebsiella pneumoniae*. All bacterial isolates were routinely maintained on Mueller-Hinton agar (HIMEDIA, India) and subcultured onto fresh nutrient agar plates as required to ensure viability and purity throughout the study. Antimicrobial susceptibility testing was assessed using disc diffusion and well-diffusion assays, following Clinical and Laboratory Standards Institute (CLSI) guidelines. Broth microdilution and Mueller-Hinton agar plating methods determined minimum inhibitory (MIC) and minimum bactericidal concentration (MBC).

##### 2.1.3.2 Inoculum standardization

Bacterial isolates were standardized to a 0.5 McFarland turbidity, approximately equivalent to ~1 × 10^8^ CFU/mL. In brief, a single loopful of the respective colony was transferred to 10 mL of sterile distilled water in a glass tube, ensuring that the optical density matched the 0.5 McFarland reference (Haley et al., 2024).

##### 2.1.3.3 Fungal culture

A clinical isolate of *Candida albicans* from the sputum of the 35-year-old male patient was obtained and cultured on Sabouraud Dextrose Agar (SDA) at 37°C. Following initial growth, the isolate was subcultured as required for antifungal evaluation.

#### 2.1.4 Antimicrobial assays

##### 2.1.4.1 Determination of MIC and MBC/MFC

The antimicrobial efficacy of the plant extracts was assessed using standardized broth microdilution and agar diffusion techniques following Clinical and Laboratory Standards Institute (CLSI) recommendations (Haley et al., 2024). Serial two-fold dilutions of each plant extract were prepared in Mueller-Hinton Broth (MHB) to obtain final 100, 50, 25, and 12.5 mg/mL concentrations. Each sterile 96-well microtiter plate received 100 μL of the diluted extract and 100 μL of a bacterial suspension standardized to a 0.5 McFarland turbidity. The following experimental controls were included to ensure assay reliability: a growth control (MHB and bacterial inoculum), a sterility control (MHB and extract without bacterial inoculum), a DMSO control (MHB, bacterial inoculum, and DMSO), and a solvent control (MHB, bacterial inoculum, and either methanol or acetone), to account for potential solvent effects. Following incubation at 37°C for 18–24 h, the minimum inhibitory concentration (MIC) was defined as the lowest concentration of extract that inhibited visible microbial growth. To determine the minimum bactericidal concentration (MBC), 10 μL aliquots from wells showing no turbidity were subcultured onto Mueller-Hinton Agar (MHA), and the lowest concentration at which no colonies were observed after incubation was considered as the MBC (Liu et al., 2017; Sánchez-Gutiérrez et al., 2021; Kebede and Shibeshi, 2022; Esfandiary et al., 2024).

For the disc diffusion assay, MHA plates were uniformly inoculated with a 0.5 McFarland bacterial suspension using sterile cotton swabs. Sterile 6 mm filter paper discs were saturated with plant extracts (100, 50, 25, and 12.5 mg/mL) prepared in DMSO or sterile water. Positive control discs were antimicrobial agents (e.g., Levofloxacin 5 μg/disc was used as an antibacterial agent, Sulconazole 25 μg disc was used as the antifungal control), while negative control discs contained only the solvent (methanol, acetone, or DMSO). After air-drying the discs for ~5 min to ensure complete solvent evaporation, the discs were aseptically placed on the surface of the inoculated plates, maintaining a minimum spacing of 24 mm. Plates were incubated at 37°C for 18–24 h, after which inhibition zones (including the disc diameter) were measured in millimeters and compared to the standard antibiotic controls (Liu et al., 2017; Sánchez-Gutiérrez et al., 2021; Kebede and Shibeshi, 2022; Esfandiary et al., 2024).

Similarly, MHA plates were inoculated in the well diffusion assay as described above. Wells of 6–8 mm diameter were aseptically made using a sterile cork borer, and 100 μL of each plant extract concentration (100, 50, 25, and 12.5 mg/mL) was loaded into individual wells. Solvent and antibiotic controls were also applied. Plates were left at room temperature for 10 min to facilitate initial diffusion, followed by incubation at 37°C for 18–24 h. The inhibition zone surrounding each well was measured and recorded (Liu et al., 2017; Sánchez-Gutiérrez et al., 2021; Kebede and Shibeshi, 2022; Esfandiary et al., 2024).

##### 2.1.4.2 Control measures

Levofloxacin (5 μg/disc) was used as the positive control for antibacterial activity, whereas Sulconazole (25 μg) was used as the antifungal control. To ensure appropriate negative controls, both the extraction solvents (methanol, ethanol, and aqueous media) and a 10% DMSO solution were tested to ensure appropriate negative controls. DMSO was included based on its frequent use as a solvent for plant extracts and bioactive compounds. However, to mitigate its potential antimicrobial effect, we ensured that the DMSO final concentration in microbial assays remained below inhibitory levels, as previously reported in the literature (Gonelimali et al., 2018; Ratananikom and Srikacha, 2020; Summer et al., 2022). Furthermore, methanol, ethanol, and aqueous media were individually tested as standalone negative controls to eliminate the possibility of solvent-induced antimicrobial effects. These tests confirmed that the extraction solvents did not exhibit antimicrobial activity at the concentrations used in the experiments, validating their appropriateness as controls.

#### 2.1.5 *In vitro* reporter-gene profiling of toxicity pathways

We employed toxicological assay software (Attagene, Inc., Morrisville, NC, USA) to evaluate critical toxicological endpoints encompassing nuclear receptor signaling pathways, stress response pathways, molecular initiating events (MIEs), and metabolism. Unless otherwise noted, all procedures were performed according to the manufacturer's instructions (which included guidelines for plate preparation, reagent handling, incubation conditions, and normalization of data). Although the Attagene platform employs automated software analytics, the TF-CIS/NRF assay is a cell reporter-gene *in vitro* screening system; therefore, all pathway data reported here originate from cultured HepG2-derived cells rather than purely computational (*in silico*) simulations. We evaluated three plant extracts*: O*. *europaea* methanolic fraction, *O. europaea* aqueous fraction, and *F. carica* methanolic fraction. Each extract was tested at five concentrations: 6.25, 12.5, 25, 50, and 100 mg/mL, with 0.5% DMSO as the constant vehicle.

The Tox21 software was used to determine the activity of the following nuclear receptor signaling pathways: aryl hydrocarbon receptor (AhR), androgen receptor (AR), androgen receptor ligand binding domain (AR-LBD), aromatase, estrogen receptor alpha (ER), and estrogen receptor ligand binding domain (ER-LBD), and peroxisome proliferator-activated receptor gamma (PPAR-Gamma). Using the same platform, stress response pathways were detected by assessing the activity of key stress-related factors: nuclear factor (erythroid-derived 2)-like 2/antioxidant responsive element (nrf2/ARE); heat shock factor response element (HSE); mitochondrial membrane potential (MMP); phosphoprotein (tumor suppressor) p53; and ATPase family AAA domain-containing protein 5 (ATAD5). Reporter cells or assays specific to each stress factor were employed according to the manufacturer's guidelines, which included protocol-driven cell seeding, compound exposure, and post-treatment incubation. The resulting signals were detected and analyzed according to the software's instructions. Positive-control agonists for each pathway (e.g., TCDD for AhR, 17β-estradiol for ERα, rosiglitazone for PPAR-γ, and tBHQ for Nrf2/ARE) were run in parallel to validate assay performance. At the same time, vehicle-only wells served as baselines for fold-induction calculations.

To further identify molecular initiating events, the assay software was further applied to measure the activity of the following receptors and enzymes: thyroid hormone receptor alpha (THRα), thyroid hormone receptor beta (THRβ), transthyretin (TTR), ryanodine receptor (RYR), GABA receptor (GABAR), glutamate N-methyl-D-aspartate receptor (NMDAR), alpha-amino-3-hydroxy-5-methyl-4-isoxazole-propionate receptor (AMPAR), kainate receptor (KAR), acetylcholinesterase (AChE), constitutive androstane receptor (CAR), pregnane X receptor (PXR), NADH-quinone oxidoreductase (NADHOX), voltage-gated sodium channel (VGSC), and Na^+^/I^−^ symporter (NIS). We also expand our investigation using the Attagene platform to assess metabolic activity by measuring the activity of the following cytochrome P450 enzymes: cytochrome CYP1A2, CYP2C19, CYP2C9, CYP2D6, CYP3A4, and CYP2E1. A reporter was classified as “active” when the fold-induction exceeded 1.5 × vehicle control in at least two adjacent concentrations and demonstrated a concentration-dependent trend with Hill-fit *R*^2^ ≥ 0.9; otherwise, it was deemed inactive.

### 2.2 *In vivo* studies

#### 2.2.1 Animal model

All animal experiments were carried out in accordance with institutional guidelines and international regulations for the care and use of laboratory animals, following the ARRIVE 2.0 guidelines (Percie du Sert et al., 2020). Fifteen healthy male BALB/c mice were purchased from the University of Benghazi, Faculty of Medicine Animal House. Individual mice were 1–2 months old, with an average weight of 25–30 g. Animals were maintained at a 12-h light/dark cycle at 24–25°C, receiving unrestricted access to tap water and a standard commercial rodent diet throughout both the acclimation and experimental phases. The mice were randomly assigned to three experimental groups (*n* = 5 per group). The selection of five mice per group was guided by considerations of scientific rigor, ethical responsibility, and consistency with prior *in vivo* studies. A sample size of *n* = 5 is commonly employed in murine models investigating plant extracts, as it provides adequate statistical power while reducing excessive animal use (Charan and Kantharia, 2013). Moreover, this selection aligns with the principles of Reduction, Replacement, and Refinement (3Rs), ensuring ethical compliance without compromising data integrity. Moreover, established guidelines in experimental design recommend similar sample sizes for preclinical studies to balance statistical robustness and ethical responsibility (Festing and Altman, 2002; Piper et al., 2022). The first experimental group was administered intraperitoneal injections with the methanolic fraction of olive leaf extract, reconstituted in distilled water at 50 mg/kg. The second group received the same extract at 100 mg/kg dose. The control group was administered distilled water. For *in vivo* administration, the methanolic extract of *O. europaea* was reconstituted in distilled water to ensure biocompatibility and reduce solvent-related toxicity in accordance with ethical and safety standards for animal research. The doses of 50 and 100 mg/kg of *O. europaea* leaf extract were selected based on previous studies demonstrating their safety and efficacy in animal models. Oral administration of olive leaf extracts at doses up to 200 mg/kg has shown therapeutic effects without toxicity, while acute toxicity studies report no adverse effects at doses up to 2,000 mg/kg (Clewell et al., 2015; Guex et al., 2018; Hinad et al., 2021). Before sacrifice, animals were fasted but retained free access to water until 24 h before euthanasia. *In vivo* experiments were conducted exclusively with the methanolic extract of olive leaves due to its promising bioactivity observed in preliminary *in vitro* evaluations, thus warranting further investigation. In contrast, fig leaf extract was excluded from *in vivo* testing due to practical limitations, such as restricted laboratory animal availability and the necessity to optimize resource allocation to ensure a statistically significant and ethically compliant study design. All procedures were carried out in accordance with institutional guidelines for animal care and use and conformed to internationally recognized ethical standards.

#### 2.2.2 Ethical approval

All clinical samples were anonymized prior to analysis to ensure patient confidentiality. The study was approved by the Institutional Ethics Review Board and conducted in accordance with the ethical principles outlined in the Declaration of Helsinki (approval reference number: LIMU/UMC/IRB/2024-027). The Institutional Animal Care and Use Committee, University of Benghazi, reviewed and approved animal protocols (animal ethical approval certificate number: IACUCB-MED-JY/2024/015). All experimental protocols were performed in accordance with institutional guidelines for the care and use of laboratory animals.

#### 2.2.3 Histological techniques and sample collection

The histological methods employed in this study were conducted to assess potential tissue alterations induced by olive and fig leaf extracts, detect cellular and inflammatory responses, evaluate safety and adverse effects, and provide microscopic evidence of physiological impacts not detected by biochemical assays alone.

##### 2.2.3.1 Organ removal and fixation

Mice were anesthetized by diethyl ether inhalation 24 h after the injection of olive leaf extracts. These animals were then euthanized, and liver (hepatectomy) and kidney (nephrectomy) tissues were extracted in aseptic conditions. Each tissue sample was placed in 10% neutral-buffered formalin for 24 h to preserve the structure. After fixation for the first time, standard histological preparations were performed as described by Suvarna et al. (2020). Briefly, samples remained in 10% formalin to stabilize tissue architecture, and tissues were sequentially exposed to increasing ethanol concentrations (50%, 70%, 90%, and 100%) to remove residual water. Dehydrated samples were immersed in xylene for ~2 h to replace ethanol and increase tissue transparency. Tissues were infiltrated with molten paraffin wax, providing mechanical support and enabling thin sectioning, and transferred to metallic or plastic molds, which were then rapidly cooled (−6 to −4°C) to solidify the wax block, thereby fixing the tissue orientation. Blocks were trimmed into 3–5 μm sections with a rotary microtome for sectioning. Ribbon sections were floated on a 40–45°C water bath and subsequently mounted onto microscope slides pre-treated with an adhesive or 50% ethanol to facilitate adherence. The slides were then dried before staining 0.50% ethanol or adhesive is needed to perform well, and the slides were dried before staining.

##### 2.2.3.2 Hematoxylin and eosin staining

The staining procedure was conducted according to the method described by Fischer et al. (2008), with minor modifications. For deparaffinization and rehydration, the slides were placed in xylene for about 2 min, then in descending concentrations of ethanol (96%, 90%, 70%) to rehydrate the tissue sections. Slides were then immersed in hematoxylin solution for 5 min, rinsed under running water, briefly dipped in acid alcohol (one dip) for differentiation, and thoroughly rewashed. These sections were transferred to eosin for 3 min, then lightly rinsed in water to remove excess stain and dehydrated for mounting. The slides were finally immersed in xylene to clear residual ethanol and mounted with DPX (styrene plasticizer xylene). A coverslip was gently lowered to seal the tissue sections.

##### 2.2.3.3 Histopathological evaluation

Prepared slides were examined under an Olympus BX41 light microscope. Images were captured digitally with an Olympus DP2-BSW system. Pathological assessments focused on identifying inflammatory lesions, necrosis, and other cellular or architectural alterations relevant to olive leaf extract treatment effects.

##### 2.2.3.4 Hematological and biochemical analysis

Blood samples were drawn via puncture using a 30-gauge needle. A portion of the blood was collected into EDTA tubes for complete blood count (CBC) analysis using a SYSMEX XN330 (Diamond Diagnostics, USA) hematology analyzer, determining red blood cells (RBCs), white blood cells (WBCs), hemoglobin, and platelets. The remaining blood sample was placed into plain tubes centrifuged at 4,000 rpm, and the resulting serum was evaluated for liver and kidney function using a Cobas Integra 400 plus platform (Roche Diagnostics, Germany). Parameters such as alanine transaminase (ALT), aspartate transaminase (AST), creatinine, and urea were measured to assess organ function (Arantes-Rodrigues et al., 2011; Clewell et al., 2015).

### 2.3 *In silico* studies

#### 2.3.1 Molecular docking studies

**Figure 1:**
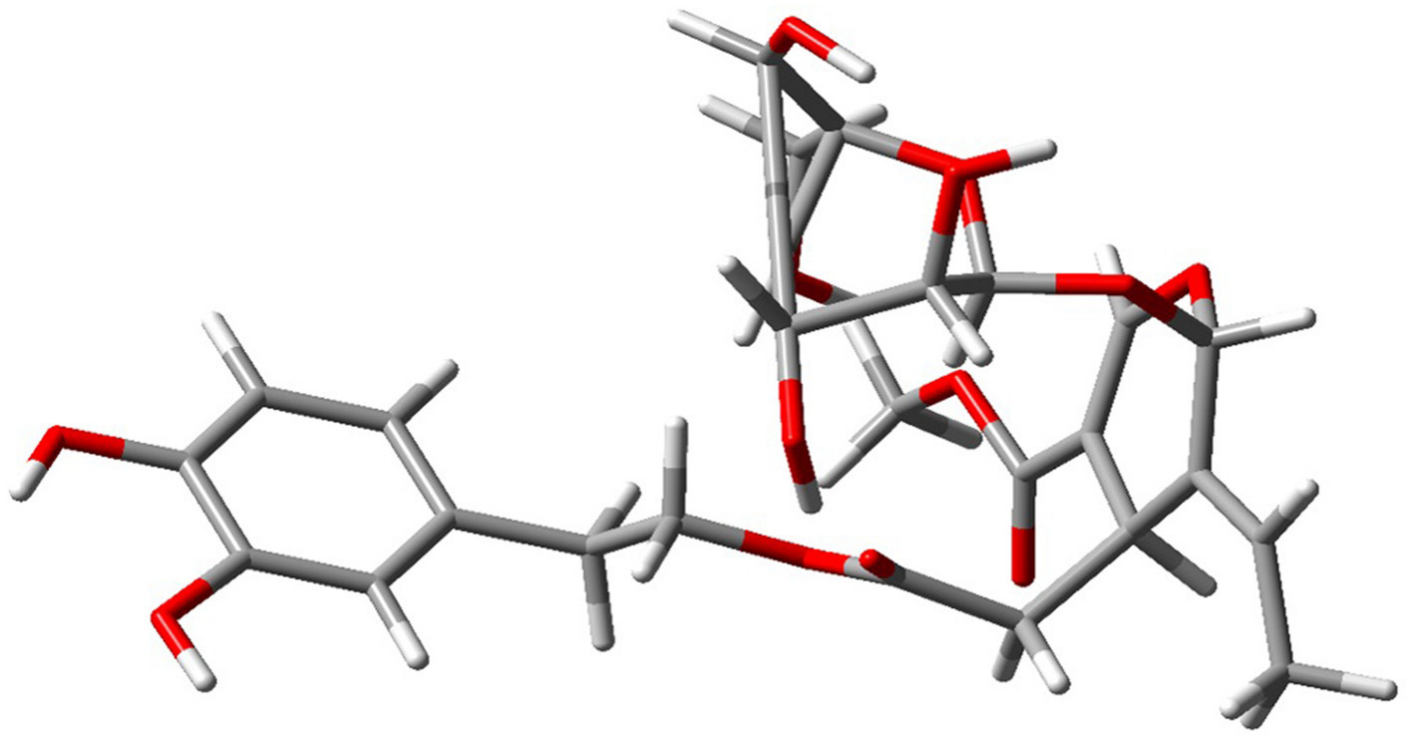
Optimized minimum-energy geometry of a representative olive leaf extract compound obtained using DFT calculations [B3LYP/6-311++G(d,p)] in Gaussian 09. The absence of imaginary vibrational frequencies in the vibrational analysis confirms that the structure corresponds to an actual local minimum on the potential energy surface.

**Figure 2:**
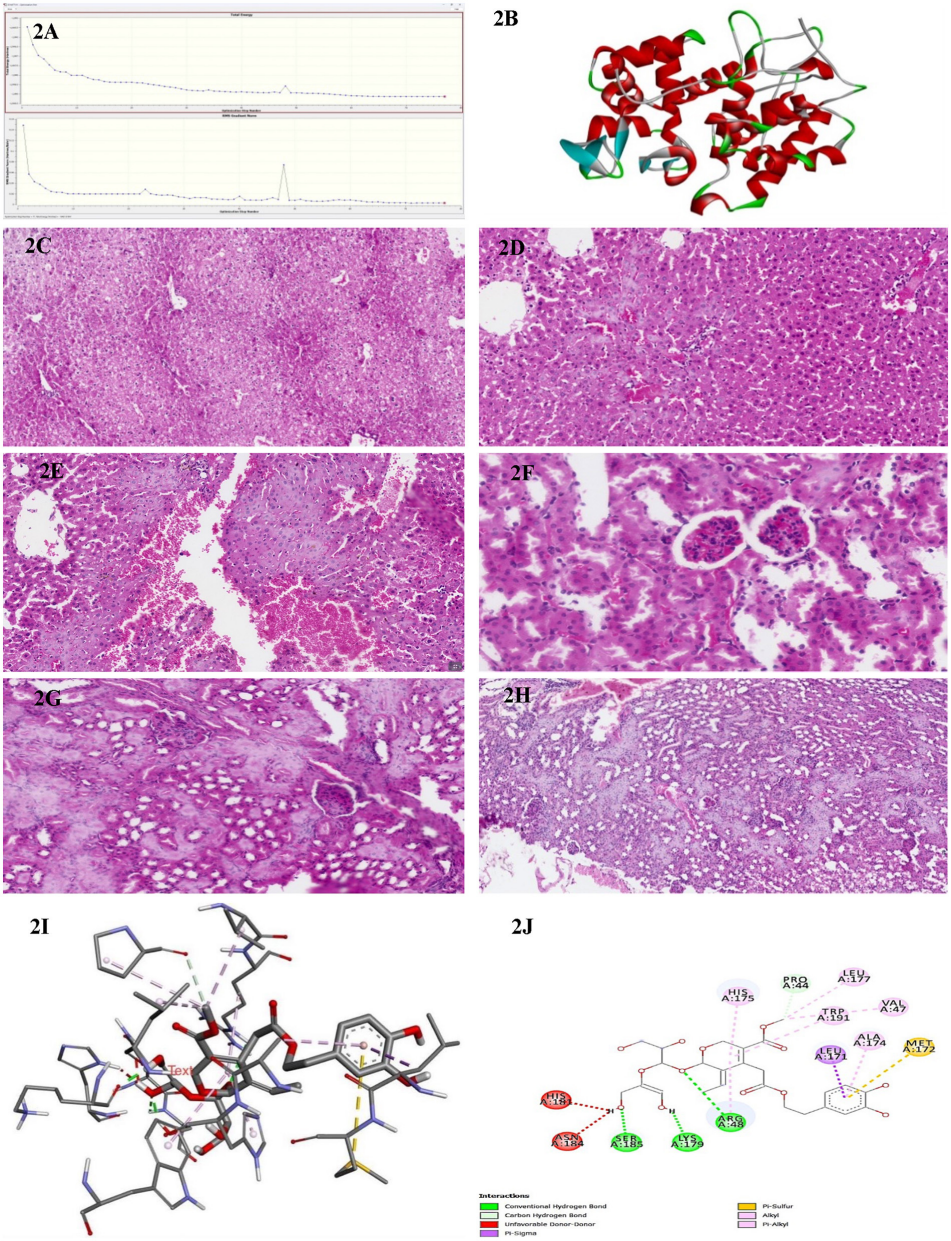
**(A)** Energy convergence diagram obtained during the DFT [B3LYP/6-311G++(d,p)] geometry optimization of olive leaf extract in Gaussian 09. The top panel shows the total electronic energy at each optimization step, while the bottom panel depicts the root-mean-square (RMS) gradient norm. The systematic decrease in energy and gradient norm over successive iterations indicates successful convergence to a stable local minimum on the potential energy surface. **(B)** Three-dimensional structure representation of the peroxidase enzyme from *E. coli* (PDB ID: 1BEK). Alpha-helices are depicted in red, β-sheets in cyan, and loop regions in green. This structure was obtained from the Protein Data Bank and visualized here to illustrate the overall fold and secondary structural elements characteristic of bacterial peroxidases. **(C)** A representative histological section of the liver from a BALB/c control mouse administered an intraperitoneal injection of distilled water, stained with hematoxylin and eosin, and examined at 200 × magnification. **(D)** A representative histological section of the liver from a BALB/c mouse administered an intraperitoneal dose of 50 mg/kg of the methanolic fraction of olive leaf extract reconstituted in distilled water. The tissue was stained with hematoxylin and eosin and examined at 200 × magnification. **(E)** A representative histological section of the liver from a BALB/c mouse administered an intraperitoneal dose of 100 mg/kg of the methanolic fraction of olive leaf extract reconstituted in distilled water. The tissue was stained with hematoxylin and eosin and examined at 200 × magnification. **(F)** A representative histological section of the kidney from a BALB/c control mouse was administered an intraperitoneal injection of distilled water. The tissue was stained with hematoxylin and eosin and examined at 200 × magnification. **(G)** A representative histological section of the kidney from a BALB/c mouse following intraperitoneal injection with 50 mg/kg of the methanolic fraction of olive leaf extract reconstituted in distilled water. The tissue was stained with hematoxylin and eosin and observed at 200 × magnification. The section exhibits cortical hemorrhages and focal regions of interstitial damage. **(H)** A representative histological section of the kidney from a BALB/c mouse following intraperitoneal injection with 100 mg/kg of the methanolic fraction of olive leaf extract reconstituted in distilled water. The tissue was stained with hematoxylin and eosin and observed at 200 × magnification. The section exhibits cortical hemorrhages and focal regions of interstitial damage. **(I)** The three-dimensional molecular docking representation of oleuropein (olive leaf extract) bound to bacterial peroxidase. The model highlights critical tight binding interactions, including hydrogen bonds and hydrophobic contacts, illustrated as dashed lines. This interaction provides insights into Oleuropein's potential mechanism of action in modulating bacterial peroxidase activity. **(J)** The two-dimensional interaction map of Oleuropein (olive leaf extract) with bacterial peroxidase highlights the key molecular interactions. The diagram illustrates hydrogen bonds (green and red dashed lines), carbon-hydrogen bonds, and various hydrophobic interactions, such as π-alkyl, π-sulfur, and π-π stacking, which contribute to the binding stability. Residues involved in these interactions are labeled, providing a detailed view of the compound's binding orientation and affinity for peroxidase.

All calculations were initiated by preparing and optimizing the ligand as *O. europaea* leaf extract with Gaussian 09 (Gaussian Inc., Wallingford, CT, USA). The quantum-chemical geometry optimizations used DFT at the B3LYP functional level with the 6-311 G++(d,p) basis set, a commonly used combination where computational load and accuracy are balanced. Subsequent vibrational-frequency analyses were conducted to ensure no imaginary frequencies were present, confirming that optimized structures corresponded to true potential-energy minima. The energy diagram (Figure 1) and the refined version of the final geometry (Figure 2A) were exported from Gaussian 09 to ensure reproducibility and clarity.

We selected the cytochrome-c peroxidase (Ccp) because it is an essential periplasmic H_2_O_2_-detoxifying enzyme in many of the Gram-negative MDR pathogens examined in this study. Chemical or genetic inhibition of Ccp markedly increases oxidative-stress sensitivity and attenuates virulence. The high-resolution *E. coli* Ccp structure (PDB ID 1BEK, 1.8 Å) provides a conserved, biologically relevant, and readily druggable target for our panel of Enterobacteriaceae isolates. The *E. coli* peroxidase structure was obtained from the Protein Data Bank (PDB ID: 1BEK; https://www.rcsb.org) and processed to remove any unnecessary entities, including crystallographic water and non-essential cofactors, keeping only the functional portion of the enzyme-binding site. Missing hydrogen atoms were added, and side-chain orientations were validated using the standard protein-preparation protocols. PyMOL (v 2.0; Schrödinger, LLC) was used to visualize the α-helices, β-sheets and loop regions in red, cyan, and green, respectively (Figure 2B). This color scheme and rendering style were selected to emphasize the overall fold of the protein with a focus on the structural motifs essential for enzymatic catalysis.

For the molecular docking analyses, 10 phenolic compounds were selected based on their consistent identification as major constituents in methanolic leaf extracts through recent LC-MS and HPLC-MS studies. These included oleuropein, hydroxytyrosol, tyrosol, verbascoside, luteolin-7-O-glucoside, and apigenin from *Olea europaea* (Kabbash et al., 2023; Papageorgiou et al., 2022), as well as psoralen, eugenol, acetyleugenol, and quercetin from *Ficus carica* (Kim and Lee, 2023; Tikent et al., 2024; Shiraishi et al., 2023). These compounds were selected for docking based on their well-documented antimicrobial and redox-modulatory properties, adherence to Lipinski's and Veber's criteria for drug-likeness, their substantial representation in the methanolic extract (≥5% of the total peak area), and the availability of high-quality, structurally resolved 3D coordinates enabling robust computational modeling.

Pre-docking and docking simulations were conducted using established molecular-docking software, such as AutoDock Vina and Schrödinger Glide (MOE-Dock). The optimized olive-leaf-extract ligands were converted into the appropriate docking-file formats (PDBQT) suitable for each software package. During ligand preparation, rotatable bonds, such as broken rings, were carefully defined to ensure accurate modeling of ligand flexibility. Reported catalytic residues were combined with automated pocket-detection algorithms for peroxidase active-site delineation. Multiple independent docking runs allowed for extensive exploration of the conformational landscape, resulting in various plausible ligand poses. We further examined the high-scoring solutions in detail, with a focus on hydrogen-bond formation, π-π stacking, hydrophobic contacts, and potential electrostatic clashes, particularly for functionally important residues (e.g., Ser 185, Lys 179, Arg 48).

### 2.4 Statistical analysis

All data were processed using Microsoft Excel 2023 and are expressed as means ± standard deviation (SD). Statistical significance across multiple experimental groups was assessed via one-way analysis of variance (ANOVA) with repeated measures, utilizing SPSS software (SPSS, Inc., Chicago, IL). Additionally, a *t*-test was conducted to determine differences between two independent group means. Furthermore, two-way ANOVA was employed to simultaneously evaluate the influence and interactions of two independent factors, providing a more comprehensive understanding of their combined effects on the results. Differences with *p*-values below 0.05 (*p* < 0.05) were considered statistically significant.

## 3 Results

### 3.1 *In vitro* studies

#### 3.1.1 Antimicrobial susceptibility testing of the clinical isolates

We evaluated antimicrobial susceptibility profiles for the selected clinically relevant pathogens against a panel of antimicrobial agents, as shown in Table 1. Our results revealed that *E. faecium* exhibited complete resistance to all tested antimicrobial agents, suggesting a pan-resistant phenotype. *K. pneumoniae* exhibited extensive antimicrobial resistance but retained susceptibility to amoxicillin-clavulanate and ceftazidime. In contrast, *S. aureus* was sensitive to azithromycin, nitrofurantoin/fosfomycin, trimethoprim-sulfamethoxazole, and amikacin but resistant to gentamicin, vancomycin, amoxicillin-clavulanate, ceftriaxone, ceftazidime, and tobramycin. Furthermore, *E. coli* was susceptible to azithromycin, amikacin, and gentamicin but resistant to nitrofurantoin/Fosfomycin, trimethoprim-sulfamethoxazole, vancomycin, amoxicillin-clavulanate, ceftriaxone, ceftazidime, and tobramycin. *E. faecalis* exhibited a limited antimicrobial susceptibility profile, demonstrating sensitivity exclusively to trimethoprim-sulfamethoxazole while exhibiting resistance to all other agents tested. *S. agalactiae* exhibited complete resistance to all antimicrobial agents tested. *E. cloacae* remained resistant to azithromycin and amoxicillin-clavulanate but susceptible to trimethoprim-sulfamethoxazole and amikacin. Two-way ANOVA revealed statistically significant differences in antimicrobial agents efficacy (*p* = 0.042). These findings highlight the widespread occurrence of multidrug resistance in specific microbial pathogens, particularly *E. faecium* and *S. agalactiae*. Furthermore, the results emphasize the necessity of ongoing monitoring, careful antimicrobial agent selection, and responsible antimicrobial practices.

**Table 1:** Zone of inhibition diameters (in mm, presented as mean ± standard deviation) for various clinically relevant pathogens tested against a panel of antimicrobial agents.

**Pathogen**	**AZM**	**NI/F**	**SXT**	**AK**	**CN**	**VA**	**AMC**	**CRO**	**CAZ**	**TOP**
*E. faecium*	17.00, 1.00	10.67, 0.57	8.33, 0.57	15.00, 1.00	16., 0.57	9.33, 0.57	9.33, 1.15	8.67, 0.57	9.00, 1.00	9.33, 1.52
*K. pneumoniae*	17.00, 1.00	9.66, 1.15	10.66, 0.57	10.66, 0.57	9.66, 1.52	8.66, 0.57	27.34, 1.15	10.33, 1.15	26.00, 0.00	8.33, 1.15
*S. aureus*	32.00, 1.00	30.33, 1.155	30.66, 0.57	26.66, 0.57	10.00, 1.00	11.00, 1.00	11.000, 1.00	9.66, 0.57	9.00, 1.00	9.66, 0.57
*E. coli*	30.33, 1.15	10.66, 0.57	10.33, 0.57	25.66, 0.57	26.00, 0.00	9.66, 0.57	9.33, 1.15	13.66, 0.57	10.66, 1.52	9.33, 1.15
*E. faecalis*	12.66, 1.15	8.66, 0.57	33.33, 1.155	16.33, 0.57	17.66, 0.57	11.33, 0.57	8.66, 0.57	10.33, 0.57	9.33, 0.57	11.66, 0.57
*S. agalactiae*	10.33, 0.57	9.66, 0.57	11.00, 1.00	11.33, 0.57	11.00, 1.00	11.67, 1.15	12.67, 0.57	11.67, 0.57	9.67, 0.57	10.00, 1.00
*E. cloacae*	10.33, 0.57	10.00, 0.00	32.00, 1.00	38.00, 1.73	9.67, 0.57	6.00, 0.00	12.67, 0.57	10.00, 1.00	6.00, 0.00	6.00, 0.00

Measurements were obtained following the Clinical and Laboratory Standards Institute (CLSI) guidelines. AZM, Azithromycin; NI/F, Nitrofurantoin; SXT, Trimethoprim/Sulfamethoxazole; AK, Amikacin; CN, Gentamicin; VA, Vancomycin; AMC, Amoxicillin/Clavulanic Acid; CRO, Ceftriaxone; CAZ, Ceftazidime; and TOP, Tobramycin.

#### 3.1.2 Antimicrobial efficacy of *O. europaea* and *F. carica* leaf extracts using different solvents

In parallel, a range of *in vitro* assays was performed on *O. europaea* and *F. carica* leaf extracts against the previously described pathogenic bacterial strains and *C. albicans*. Extracts were prepared using three different solvents (acetone, methanol, and distilled water) at five concentrations (100, 50, 25, 12.5, 6.25 mg/mL). Antimicrobial activity was assessed using the agar well diffusion assay, with inhibition zones recorded in millimeters from three independent replicates. To ensure the specificity of the antimicrobial activity, each solvent alone was tested in parallel with the target pathogens as a solvent control. The negative control consisted of 10% DMSO, while standard antimicrobial agents were positive controls.

##### 3.1.2.1 Acetone extracts

At 100 mg/mL concentration, *O. europaea* acetone extracts demonstrated moderate inhibitory effects against some bacterial isolates. The most significant susceptibility was observed in *K. pneumoniae* (14.67 mm) and *E. coli* (14.33 mm), followed by modest zones against *S. aureus* (11 mm) and *S. agalactiae* (10 mm), as shown in Supplementary Table 1. However, no inhibition was detected at lower extract concentrations, nor against *Enterococcus* spp., *P. aeruginosa*, or *C. albicans*. In contrast, *F. carica* acetone extracts displayed minimal activity. Only *K. pneumoniae* showed a small inhibition zone (8.67 mm) at 100 mg/mL, with no measurable effects against any other tested organism at lower concentrations.

These findings suggested that the antimicrobial efficacy of acetone extracts is notably limited. At the highest concentration, the extracts showed effectiveness only against a few Gram-positive and Gram-negative bacterial strains. Furthermore, neither extract demonstrated antifungal efficacy against *C. albicans*, indicating poor extraction of antifungal constituents using acetone.

Overall, both acetone extracts demonstrated narrower spectra and smaller inhibition zones relative to the positive controls (e.g., up to 32.33 mm for *K. pneumoniae*). These results suggest that acetone-based extracts exhibit limited antimicrobial efficacy, particularly at lower concentrations. Consequently, acetone appears to be a less effective solvent for extracting pharmacologically active antimicrobial constituents from these plant sources.

##### 3.1.2.2 Methanol extracts

Methanol emerged as the most effective solvent for extracting bioactive compounds from *O. europaea* (Supplementary Table 2). The methanolic extract exhibited potent antibacterial activity at the highest tested concentration (100 mg/mL), notably against *S. agalactiae* (29 mm) and *E. cloacae* (39.33 mm). Even at 50%, considerable inhibition zones were observed, particularly for *S. agalactiae* (20.67 mm) and *K. pneumoniae* (20.33 mm). A moderate antifungal effect was also evident at 100% against *C. albicans* (19.66 mm). However, moderate fungal inhibition was observed at lower concentrations.

In contrast, the methanolic extract of *F. carica* exhibited comparable antimicrobial potency. At 100 mg/mL, the extract exhibited wider inhibition zones: 20 mm for *S. aureus*, 8 mm for *K. pneumoniae*, 9.33 mm for *E. coli*, and 13.67 mm for *S. agalactiae*. At lower concentrations, the extracts exhibited minimal to no detectable antimicrobial activity, and *C. albicans* showed complete resistance across all tested concentrations.

These findings suggest that methanol is highly efficient in extracting antimicrobial phytochemicals from *O. europaea*, presumably due to its strong capacity to solubilize polar compounds such as polyphenols and flavonoids. The observed concentration-dependent activity further highlights the importance of solvent selection and dosage in optimizing the recovery of pharmacologically active constituents. The broad-spectrum efficacy of *O. europaea* methanolic extracts against multiple bacterial strains underscores its potential for further investigation as a plant-derived antimicrobial agent.

##### 3.1.2.3 Water extracts

The experimental findings revealed that at 100 mg/mL concentration, aqueous extracts of *O. europaea* exhibited moderate to potent antimicrobial activity, particularly against *E. cloacae* (23.10 mm), *S. aureus* (20.3 mm), *P. aeruginosa* (27 mm), and *E. faecium* (17.7 mm) as shown in Supplementary Table 3. *K. pneumoniae* and *E. coli* exhibited limited susceptibility to the aqueous extracts, each showing inhibition zones of 14.7 mm. In contrast, *S. agalactiae, E. faecalis*, and *C. albicans* were entirely resistant with no observable inhibitory effect. Notably, a moderate level of antimicrobial activity remained detectable at 50 and 25 mg/mL concentrations, particularly against *S. aureus* (12.7 and 7.3 mm, respectively) and *E. coli* (14.3 mm at 50%). However, no measurable inhibitory effects were observed at lower concentrations or against the remaining tested isolates. Consistent with its limited activity in other solvents, *F. carica* aqueous extracts exhibited only weaker antimicrobial activity. Inhibition was observed sporadically at 100 mg/mL, specifically against *S. aureus* (11.33 mm). However, most other bacterial isolates and *C. albicans* showed complete resistance. These findings suggest water is a suboptimal solvent for extracting bioactive antimicrobial constituents from *F. carica*. These findings suggest that although aqueous extracts of *O. europaea* display moderate antibacterial activity, their overall efficacy remains markedly lower than that of the methanolic extracts. Nevertheless, due to the absence of organic solvents, their non-toxic nature and compatibility with *in vivo* applications underscore their relevance in specific therapeutic contexts. These findings further underscore the critical importance of solvent selection in enhancing the antimicrobial efficacy and potential clinical utility of plant-based therapeutics.

In conclusion, these findings demonstrate that the antimicrobial effectiveness differs considerably based on the solvent type and the tested plant species. Methanol proved to be the most effective solvent for extracting bioactive compounds from *O. europaea*, demonstrating potent broad-spectrum antibacterial activity and moderate antifungal efficacy at the highest concentration tested. In contrast, extracts from *F. carica* exhibited limited antimicrobial potency against specific bacterial strains at the highest methanolic concentration. Moreover, aqueous extracts of *O. europaea* showed moderate antibacterial activity against particular pathogens, whereas acetone extracts exhibited more limited activity even at higher concentrations. These observations emphasize the significant role of solvent selection in effectively extracting bioactive compounds. Therefore, methanolic extracts of *O. europaea* represent a promising candidate for future antimicrobial research to combat agents-resistant pathogens.

#### 3.1.3 Comparison of plant extracts and conventional antimicrobial agents against MDR pathogens

A comparison of the antimicrobial agent susceptibility data with the current plant extract results shows several noteworthy similarities and differences. Most prominently, *E. faecium* and *S. agalactiae* exhibited pan-resistance to all conventional antimicrobial agents tested, yet both demonstrated appreciable inhibition zones in response to methanolic extracts of *O. europaea*. In contrast, S. *agalactiae*, which exhibited no susceptibility to standard antimicrobial agents, displayed an inhibition zone of up to 45 mm with *O. europaea* methanol extract at 100 mg/mL concentration, suggesting that these phytochemical constituents can inhibit pathogens. Similarly, *E. faecium*—entirely resistant under antimicrobial conventional testing—was also inhibited by *O. europaea* methanolic extracts at 100 mg/mL, whereas *F. carica* extracts remained largely ineffective.

A similar pattern was observed in the cases of *S. aureus* and *K. pneumoniae*. In our analyses, both isolates displayed variable susceptibility or partial resistance to selected antimicrobial agents but showed clear zones of inhibition upon exposure to methanolic extracts of *O. europaea*. Moreover, inhibition zone for *S. aureus* and *K. pneumoniae* at 100% concentration highlight the broad-spectrum potential of *O. europaea* under these conditions. By contrast, *F. carica* extracts displayed comparatively modest or negligible antimicrobial activity against the same organisms, particularly at lower concentrations and with acetone or water extractions. For *E. coli* which was susceptible to a limited number of standard antimicrobial agents, the methanol-based *O. europaea* extract demonstrated consistent inhibition, reflecting the partial efficacy observed in the antimicrobial drug profile. *E. cloacae* also followed a similar pattern, showing high-level inhibition with *O. europaea* methanol extracts despite exhibiting limited susceptibility to conventional antimicrobial agents.

Taken together, these comparative findings highlight some key observations. First, methanol appears most effective in extracting antimicrobial compounds from *O. europaea*, as evidenced by the larger inhibition zones against pathogens that significantly resisted conventional antimicrobial agents. Secondly, *F. carica* extracts generally showed lower activity across the tested organisms, suggesting that its extracts may lack the same potency against multidrug-resistant pathogens. The fact that certain strains were resistant to multiple standard antimicrobial agents but showed susceptibility to *O. europaea* extracts underscores the potential clinical relevance of plant-derived antimicrobials. Consequently, solvent choice (particularly methanol) and plant species selection (notably O. europaea) emerge as key factors in maximizing the inhibitory efficacy against bacterial and fungal pathogens.

#### 3.1.4 *In vitro* high-content reporter-gene profiling of toxicity pathways

Toxicity prediction using *in silico* models has gained widespread application in pharmacology and toxicology, offering a rapid and efficient means of screening compounds for potential safety concerns (Wichard, 2017). These computational tools provide notable benefits in early-stage drug development by reducing time and cost while avoiding the ethical challenges posed by animal experimentation. Increasingly, regulatory bodies are recognizing the validity of *in silico* approaches for specific evaluations; for instance, the ICH M7 guideline endorses the use of QSAR models as acceptable alternatives to laboratory testing for detecting DNA-reactive impurities (Raies and Bajic, 2016; Wichard, 2017).

Using Attagene's toxicological assay software (Attagene, Inc., Morrisville, NC, USA), we evaluated olive leaf extract at doses up to 2,000 mg/kg for potential organ-specific toxicity, effects on nuclear receptors and stress response pathways, molecular initiating events, and metabolic enzymes. As presented in Supplementary Table 4, the predictive models indicated low probabilities of hepatotoxicity (0.85), neurotoxicity (0.88), and respiratory toxicity (0.54). However, relatively moderate probabilities were observed for nephrotoxicity (0.75) and cardiotoxicity (0.77), signaling potential renal and cardiovascular concerns. Additionally, the algorithm categorized olive leaf extracts as active for immunotoxicity (0.98). Clinical toxicity (0.64) refers to the potential for adverse physiological effects, such as organ toxicity, biochemical imbalances, or systemic inflammatory responses, that could arise from prolonged exposure or high doses of the studied compound. On the other hand, nutritional toxicity (0.54) denotes the possibility of disruptions in nutrient metabolism, absorption, or utilization, which may lead to deficiencies, metabolic stress, or unintended immune modulation. In contrast, *in silico* analyses predicted no significant activity for carcinogenicity (0.79), mutagenicity (0.84), cytotoxicity (0.70), and ecotoxicity (0.70), indicating a low risk of malignancy, genotoxicity, direct cellular toxicity, or environmental hazard. However, a moderate probability of blood-brain barrier (BBB) penetration (0.52) underscores the need for further pharmacokinetic investigations to evaluate potential central nervous system (CNS) exposure.

Subsequent Tox21-focused evaluations (Supplementary Table 5) indicated no significant activity against key nuclear receptors including, the aryl hydrocarbon receptor (AHR, 0.94), androgen receptor (AR, 0.97), aromatase (0.86), estrogen receptor alpha (0.75), and PPAR-gamma (0.92), suggesting minimal potential for endocrine pathway disruption. Similarly, data presented in Supplementary Table 6 indicate that olive leaf extracts did not significantly induce activation of stress response pathways such as nrf2/ARE (0.92), heat shock factor (HSE, 0.92), mitochondrial membrane potential disruption (MMP, 0.82), or p53 (0.79). Moreover, molecular initiating event (MIE)-based analyses (Supplementary Table 7) revealed consistent inactivity across several key receptor sites, including thyroid hormone receptors (THRα and THRβ), ryanodine receptors (RYR), and key neurotransmitter-gated ion channels such as GABA, NMDAR, AMPAR, and KAR. The extracts exhibited low interaction potential with key xenobiotic-sensing receptors, including CAR and PXR. Cytochrome P450 interaction predictions (CYP1A2, CYP2C19, CYP2C9, CYP2D6, CYP3A4, and CYP2E1) also revealed a minimal likelihood of inducing significant metabolic disruptions or drug-drug interactions. The *in silico* analyses suggested that olive leaf extracts exhibit minimal or no activity across several pathways and stress response mechanisms. However, this analysis suggests possible immunotoxic, nephrotoxic, and cardiotoxic effects. Supporting these predictions, our *in vivo* hematological data (Section 3.2.2) also revealed immune-related alterations, such as decreases in white blood cells and platelet counts. These findings underscore the requirements for further investigation into the potential immunotoxic effects of the extracts. Accordingly, advanced cellular assays and targeted histopathological analyses are crucial to substantiate the computational predictions and determine whether the immunomodulatory effects of olive leaf extracts are beneficial or adverse across different dosage levels.

#### 3.1.5 Toxicological evaluation of oleuropein

Oleuropein, the principal bioactive constituent of olive leaf extracts, was evaluated using the ProTox-3 modeling platform (IUPAC Food ID) to generate a comprehensive toxicity profile. As shown in Table 2, the predicted LD50 for oleuropein is 2,000 mg/kg, classifying it within Toxicity Class 4, corresponding to low acute toxicity. This suggests that while moderate oral doses may be considered relatively safe, higher concentrations require careful consideration. *In silico* predictions also revealed low mutagenic and carcinogenic potential. These findings are consistent with the reported absence of carcinogenic and mutagenic effects associated with olive leaf extracts, as shown in Table 2. In contrast, oleuropein demonstrated a high immunotoxicity score. This suggests a potential for immunomodulatory activity, which is partially supported by our *in vivo* evidence showing alterations in leukocyte and platelet counts.

**Table 2:** The structural and physicochemical properties of oleuropein illustrate key molecular characteristics such as molecular weight, hydrogen bonding potential, polarity, and partition coefficient.

**Molecular structure of oleuropein**	**Properties of oleuropein**	**Value**
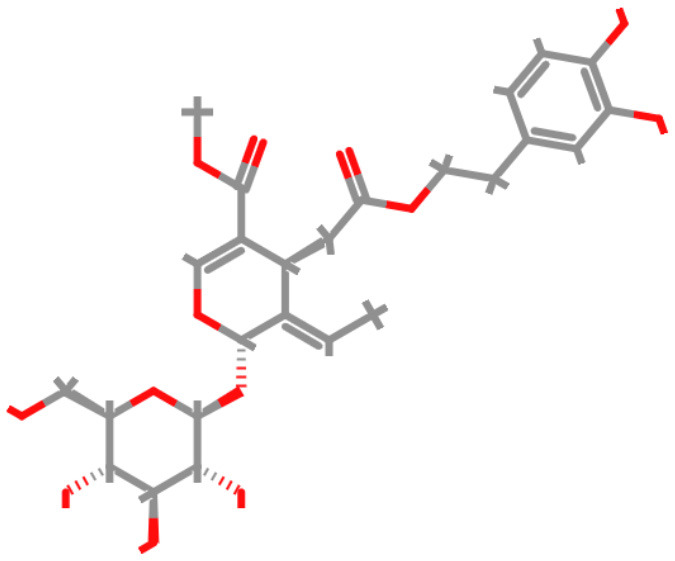	Molecular weight	540.51 g/mol
		Hydrogen bond acceptors	13
		Hydrogen bond donors	6
		Number of atoms	38
		Number of bonds	40
		Number of rotatable bonds	11
		Molecular refractivity	127.28
		Topological polar surface area	201.67 Å^2^
		Octanol/water partition coefficient (logP)	−0.63

These properties highlight the compound's chemical nature and potential interactions in biological systems. The molecular structure is shown for reference.

Mild, statistically non-significant elevations in urea levels observed in both *in vivo* biochemical analyses and *in silico* predictions suggest potential nephrotoxic effects, warranting further detailed renal investigations. In contrast, predicted low hepatotoxicity aligns with the absence of histopathological liver abnormalities in experimental animals. However, the elevation of AST levels at higher doses may indicate mild hepatic stress, highlighting the requirements for continued monitoring in long-term studies. As presented in Table 2, oleuropein's physicochemical properties-specifically its pronounced hydrophilicity (logP = −0.63) and extensive hydrogen-bonding potential (6 donors and 13 acceptors)-are likely to impact its absorption, distribution, and overall metabolic behavior. Therefore, detailed pharmacokinetic assessments are significant in accurately establishing oleuropein's safety thresholds. Evidence from our computational modeling and *in vivo* investigations underscores its potent antimicrobial and antioxidant properties. However, these benefits are accompanied by critical safety concerns, particularly regarding potential nephrotoxicity and immunotoxicity. For effective clinical translation, further investigations are essential, particularly those addressing chronic toxicity, immunophenotypic profiling, and detailed renal function assessment.

### 3.2 *In vivo* studies

#### 3.2.1 Histopathological observations

Initial histological assessment of hepatic and renal tissues from mice treated with 50 and 100 mg/kg doses revealed no overt necrosis, marked inflammation, or notable degenerative alterations. These findings correlate well with the predicted “Inactive” hepatotoxic and neurotoxic profiles from the *in silico* toxicological studies (discussed later), suggesting minimal structural compromise at the doses tested.

Microscopic sections of the liver revealed well-preserved architecture. Hepatocytes were organized in one to two thick cell plates, with uniform cytoplasm and round nuclei that were centrally located, without atypia or pleomorphism. They showed patent sinusoids and organized portal tracts containing bile ducts, portal veins, and hepatic arteries without fibrosis, inflammation, or cell infiltration. Biliary morphologic structures were also typical; the Kupffer cell population had a uniform distribution from portal to center of the lobule, and the connective tissue framework of the Globus was intact with no evidence of cirrhosis or other architectural abnormalities. Liver sections from the control group (Figure 2C) exhibited no notable signs of inflammation, necrosis, or fibrosis. In contrast, hepatic tissues from the 50 mg/kg olive leaf extract group treatment (Figure 2D) exhibited notable alterations in hepatocyte nuclear morphology and more pronounced hemorrhagic manifestations. Sections from the 100 mg/kg olive leaf extracts group treatment (Figure 2E) revealed increased hepatocellular necrosis, nuclear enlargement, and widespread hemorrhage, indicating a dose-related intensification of hepatic injury.

Renal tissues from both the right and left sides showed preserved cortical and medullary organization. The glomeruli appeared normocellular with intact capillary loops, without sclerosis or basement membrane alterations. No atrophy, degeneration, or necrosis was observed in renal tubules with normal epithelial morphology. Interstitium appeared unremarkable, while vascular structures were free of vasculitis, hyaline arteriosclerosis, or thrombosis. The collecting ducts and renal pelvic components were structurally unremarkable, with no evidence of epithelial proliferation or inflammatory changes. The kidneys from the control treatment (Figure 2F) were histologically normal aside from isolated interstitial hemorrhage. In contrast, tissues from the 50 mg/kg olive leaf extracts group (Figure 2G) exhibited more extensive interstitial hemorrhage and occasional glomerular atrophy with localized tubular atrophy. At the 100 mg/kg dose treatment, hemorrhagic changes were more prominent, accompanied by marked glomerular atrophy, suggesting a dose-related progression of renal pathology (Figure 2H).

#### 3.2.2 Hematological findings

The administration of olive leaf extract at 50 and 100 mg/kg in BALB/c mice results in several prominent changes in white blood cell (WBC) counts and platelet levels (Table 3). Both groups experienced a significant reduction in total WBC count (*p* < 0.05) compared to control, suggesting that these doses may induce an immunomodulatory response. In addition, differential leukocyte analysis showed a notable increase in lymphocyte percentages in these animal groups (*p* < 0.05), along with a corresponding decrease in neutrophils (*p* < 0.05). This shift could signify alterations in immune cell populations caused by olive leaf extract. A slight but statistically significant increase in basophils was also observed in BALB/c mice administered with 100 mg/kg dose (*p*<*0.05*).

**Table 3:** Hematological parameters in BALB/c mice (*n* = 5) following intraperitoneal administration of the methanolic fraction of olive leaf extract, reconstituted in distilled water, at doses of 50 and 100 mg/kg, compared to a control group administered distilled water.

**Parameter**	**Control**	**Group A (50 mg/kg)**	**Group B (100 mg/kg)**
WBC	8.02 ± (0.45)	6.48 ± (0.73)^*^	5.52 ± (0.41)^*^
Neutrophils	20.17 ± (0.90)	12.17 ± (1.59)^*^	15.40 ± (0.61)^*^
Eosinophils	1.57 ± (0.55)	0.97 ± (0.59)	1.30 ± (0.26)
Basophils	0.33 ± (0.25)	0.50 ± (0.53)	1.63 ± (0.25)^*^
Lymphocyte	24.63 ± (2.28)	42.23 ± (2.25)^*^	62.07 ± (10.94)^*^
Monocyte	5.57 ± (0.50)	4.47 ± (0.35)	6.23 ± (1.33)
RBC	6.37 ± (0.50)	7.82 ± (2.19)	5.07 ± (2.40)
HGB	11.63 ± (1.53)	13.98 ± (2.15)	9.63 ± (5.23)
HTC	43.70 ± (3.20)	44.70 ± (4.10)	11.90 ± (0.72)^*^
MCV	56.23 ± (3.49)	51.74 ± (2.99)	50.17 ± (1.85)
MCH	12.10 ± (0.61)	16.63 ± (1.33)^*^	15.43 ± (0.25)^*^
MCHC	30.67 ± (0.57)	31.70 ± (2.55)	33.17 ± (0.68)
Platelet	373.33 ± (40.41)	157.00 ± (8.19)^*^	213.33 ± (20.82)^*^

Data are presented as mean ± standard deviation (SD). Asterisks indicate statistically significant differences relative to the control group (p < 0.05).

Regarding red blood cell (RBC) parameters, neither treatment group exhibited a significant change in RBC count or hemoglobin (HGB) compared to controls (*p* > 0.05). However, according to *T*-test comparisons, Group B mice showed a pronounced reduction in hematocrit (HTC; *p* < 0.05). The mean corpuscular volume (MCV), mean corpuscular hemoglobin (MCH), and mean corpuscular hemoglobin concentration (MCHC) values remained within their normal ranges. Nevertheless, there was a minor yet significant rise in MCH among both treated groups (*p* < 0.05). Alterations in RBC parameters may suggest altered erythropoiesis or RBC turnover, but no clear pattern of dose-related toxicity was evident.

In both treated groups, platelet counts significantly declined (*p* < 0.05) compared to control animals, suggesting either reduced platelet production in the bone marrow or enhanced peripheral consumption (e.g., destruction or sequestration). These hematological changes highlight the importance of further investigations, including bone marrow histopathology and broader immunological evaluations, to detect whether olive leaf extract exerts immunomodulatory effects or specifically targets thrombocytes.

#### 3.2.3 Biochemical findings

Biochemical analyses revealed several notable alterations in hepatic and renal biomarkers following the administration of aqueous *Olea europaea* leaf extract (Table 4). A significant elevation in AST (Aspartate Aminotransferase) was observed in Group B (100 mg/kg), potentially suggesting possible hepatocellular injury or muscular stress associated with higher olive leaf extract exposure. Meanwhile, ALT (Alanine Aminotransferase) in the same group displayed a slight, though statistically non-significant, increase, suggesting possible hepatic involvement but no definitive proof of liver injury. *T*-test analysis also indicated that Alkaline Phosphatase (ALP) was moderately lower in Group B (*p* = 0.085) than in controls, suggesting a potential regulatory shift in liver or bone enzyme production. Animals in both Groups A and B exhibited moderate increases in urea levels; however, these changes did not reach statistical significance (*p* > 0.05). Creatinine and blood urea nitrogen (B.U.N.) remained stable across all experimental groups, suggesting minimal or no kidney impairment under the examined conditions. Furthermore, direct and indirect bilirubin levels were unaffected, implying that bilirubin metabolism remained intact. Although the slight elevation in urea may suggest mild renal or metabolic disturbances, the lack of associated changes in B.U.N. and creatinine makes definitive conclusions challenging. Therefore, additional histopathological and mechanistic studies are required to validate these findings and determine the long-term safety profile of olive leaf extract.

**Table 4:** Biochemical parameters in BALB/c mice (*n* = 5) following intraperitoneal administration of the methanolic fraction of olive leaf extract, reconstituted in distilled water, at doses of 50 and 100 mg/kg, compared to a control group receiving distilled water.

**Parameters**	**Control**	**Group A (50 mg/kg)**	**Group B (100 mg/kg)**
Urea	40.67 ± (13.03)	55 ± (21.9)	61.7 ± (6.9)
B.U.N	52.43 ± (2.58)	41 ± (7.1)	53.8 ± (3.1)
Creatinine	0.41 ± (0.13)	0.4 ± (0.1)	0.2 ± (0.1)
ALT	28.83 ± (1.24)	24.1 ± (2.7)	35.7 ± (3.2)
AST	56.25 ± (1.15)	74.9 ± (16.5)	91.4 ± (5.4)^*^
ALP	22.15 ± (1.78)	4.0 ± (0.8)^*^	2.5 ± (1.3)^*^
BIL IND	0	0	0.1
BIL DIR	0	0	0.1

Data are presented as mean ± standard deviation (SD). Asterisks denote statistically significant differences compared to the control group (p < 0.05). B.U.N., blood urea nitrogen; ALT, alanine aminotransferase; AST, aspartate aminotransferase; ALP, alkaline phosphatase; BIL IND, indirect bilirubin; BIL DIR, direct bilirubin.

### 3.3 *In silico* studies

#### 3.3.1 Molecular docking results

Molecular docking simulations targeting bacterial peroxidase (Figure 2I) identified robust non-covalent interactions between olive leaf extract constituents and the enzyme. These interactions included classical hydrogen bonding with Ser185, Lys179, and Arg48 and carbon-hydrogen bonding involving Pro44. Donor-donor repulsion observed at His181 and Asn184 suggested potential steric or electronic interference. At the same time, π-σ interactions with Leu171 and π-sulfur contacts at Met172 further characterized the complex binding architecture at the active site (Figure 2J). This intricate binding network likely contributes to the pronounced antibacterial activity associated with methanolic olive leaf extracts. It highlights the critical role of solvent choice in maximizing phytochemical recovery and bioactivity.

Although this study identified limited antifungal activity, the docking analyses provide evidence for an antibacterial mechanism involving peroxidase inhibition or disrupting bacterial redox homeostasis. Although *in silico* toxicity assessments (Supplementary Table 4) suggest potential immunotoxic risks, they highlight the requirement for cautious dosing and further mechanistic investigations to differentiate between desirable immunomodulatory effects and unintended immune suppression or hyperactivation. Future investigations should incorporate integrated pharmacokinetic and pharmacodynamic assessments alongside advanced omics-driven approaches to elucidate the comprehensive therapeutic potential of olive leaf extracts in addressing MDR pathogens.

## 4 Discussion

This study presents novel findings on the antimicrobial efficacy of *O. europaea* and *F. carica* leaf extracts against MDR pathogens. Notably, *O. europaea* leaf extracts, particularly the methanolic fraction, demonstrated potent inhibitory activity against several MDR strains, including *E. faecium* and *S. agalactiae*, which exhibited high levels of resistance to conventional antimicrobial agents. This inhibitory effect is consistent with previous reports emphasizing the potency of olive-derived phenolics, including oleuropein hydroxytyrosol and rutin, in inhibiting both Gram-positive and Gram-negative bacteria (Sudjana et al., 2009; Lee and Lee, 2010; Zorić and Kosalec, 2022). Interestingly, our findings revealed that even pan-resistant pathogens exhibited susceptibility to these polyphenolic compounds, supporting existing evidence that plant-derived antimicrobials may act through alternative mechanisms such as disrupting membrane integrity, modulating redox homeostasis, or impairing stress-response pathways (Borjan et al., 2020). The antimicrobial mechanism of olive leaf extracts against Gram-positive and Gram-negative pathogens resembles that of other plant-derived biomolecules such as green tea, oregano, and thyme, which typically exert their effects by inducing oxidative stress or disrupting microbial membrane integrity (Magyari-Pavel et al., 2024).

In contrast, the *F. carica* leaf extracts investigated in this study showed comparatively modest inhibition zones, consistent with reports suggesting that fig preparations may exhibit reduced antimicrobial activity depending on the solvent used and the extraction method (Rahmani and Aldebasi, 2017; Abdel-Rahman et al., 2021). The antimicrobial activity observed in *F. carica* extracts has been attributed to phenolic acids such as caftaric, gallic, and quercetin (Abdel-Aziz et al., 2020). Previous studies have indicated that the antimicrobial efficacy of *F. carica* may vary depending on the specific plant part utilized and the extraction solvent employed, suggesting that fig leaves contain a diverse array of bioactive compounds whose activity is significantly influenced by the extraction methodology (Abubakar and Haque, 2020). The discrepancies between our results and those reported in previous studies underscore the critical influence of variables such as solvent polarity, chemical composition, extraction temperature, and extract concentration on the antimicrobial efficacy of plant-derived bioactive compounds (Papageorgiou et al., 2022; Barolo et al., 2023; Agatonovic-Kustrin et al., 2023). Our findings are consistent with the expanding body of research on phytochemicals, including those derived from *Nigella sativa, Cinnamomum verum*, and other phenolic-rich plants (Papageorgiou et al., 2022). Furthermore, studies on green tea catechins and oregano oils suggested that combining plant phenolics with conventional antimicrobial agents may exert synergistic effects that enable dose reduction while minimizing toxicity-related concerns (Somerville et al., 2019). Regarding *F. carica*, the present findings may support the development of synergy approaches by refining extraction techniques or using combined formulations to enhance its antimicrobial potency.

Consistent with our findings, a broad range of *in vivo* and *in vitro* studies suggested that olive leaf extract is generally safe at low doses. Oleuropein, the predominant phenolic constituent of the olive leaf, has not produced adverse or low effects in animal models, even at doses as high as 1,000 mg/kg (Gonzalez-Pastor et al., 2023). In addition, human clinical data further support this favorable safety profile. In a pilot study, healthy subjects consuming olive leaf extract daily for 8 weeks displayed no notable alterations in hepatic or renal biomarkers. Interestingly, the study documented a slight increase in red blood cell counts and no reports of severe adverse reactions (Kondo et al., 2023). However, adverse effects have been reported with excessive intake or prolonged administration of olive leaf extract. It has also been demonstrated that mice fed diets containing 0.7%−0.9% olive leaf extract for 6 weeks exhibited significant elevations in liver enzyme activity and bilirubin levels, accompanied by histopathological evidence of hepatocellular vacuolation and focal necrosis (Omer et al., 2012). Moreover, prolonged consumption of olive leaf extract at dietary concentrations ranging from 0.5% to 0.75% over 14 weeks resulted in hepatic alterations, including bile duct proliferation, cholestasis, inflammatory cell infiltration, and early fibrotic changes. These pathological effects were linked to mitochondrial dysfunction, reduced membrane potential, and compromised respiratory capacity. Importantly, such effects were absent at a lower dose of 0.25%, indicating a clear threshold beyond which toxicity becomes apparent (Arantes-Rodrigues et al., 2011). The evidence suggests that while olive leaf extract is generally considered safe at conventional therapeutic doses, prolonged or high-dose administration may elicit notable hepatic, renal, and hematopoietic toxicities.

A fundamental aspect of this study is integrating *in silico* toxicity predictions with *in vitro* and *in vivo* experiments to evaluate the efficacy and safety profiles of olive leaf extracts. *In silico* approaches, including molecular docking and computational toxicity prediction, have become essential tools in antimicrobial drug discovery. They offer a rapid method of screening plant-derived compounds for potential bioactivity. Recent computational studies on *O. europaea* and *F. carica* leaf extracts illustrate the utility of these approaches. Molecular docking simulations have revealed that hydroxytyrosol, a key olive leaf phytochemical, binds to bacterial DNA gyrase and penicillin-binding protein 3 (PBP3), enzymes critical for DNA replication and cell wall synthesis (Ben Hassena et al., 2024). Notably, olive leaf phytochemicals appear to target similar pathways as conventional drugs (e.g., gyrase, a target of fluoroquinolones, and PBPs, targets of β-lactams), suggesting a mechanism for synergizing with or alternating conventional antimicrobial agents. Phenolic compounds such as oleuropein and hydroxytyrosol of olive leaves are considered safe bioactive dietary compounds. However, our computational models suggested low potential acute toxicity of *O. europaea* extracts. This finding aligns with both *in silico* toxicity assessments and *in vivo* studies consistently validating their low acute toxicity profiles (Arantes-Rodrigues et al., 2011; Guex et al., 2018; Wylie and Scott Merrell, 2022).

Similarly, *F. carica* leaf extracts have demonstrated promising *in silico* profiles. A recent study of Moroccan fig leaves identified several bioactive constituents with strong predicted binding affinities to microbial targets, including bacterial β-ketoacyl-ACP synthase (involved in fatty acid biosynthesis), nucleoside diphosphate kinase, and the fungal sterol 14α-demethylase (CYP51; Tikent et al., 2024). These findings suggest a potential for broad-spectrum antimicrobial activity, evidenced by *in vitro* activity against bacteria and *C. albicans*. Regarding safety, both experimental cytotoxicity assays and computational models suggest low to moderate toxicity, revealing selective toxicity against pathogens over host cells (Tikent et al., 2024).

Generall*y, in silico* findings of olive and fig leaf extracts follow similar patterns commonly observed in other medicinal plant investigations. Molecular docking studies consistently highlight microbial proteins such as DNA gyrase, topoisomerases, transpeptidases, and virulence regulators as common targets. For example, phytochemicals from *Azadirachta indica* (neem) and *Curcuma longa* (turmeric) have shown high binding affinities to bacterial DNA replication and quorum-sensing machinery (Wylie and Scott Merrell, 2022; Dai et al., 2022). Likewise, *Snapdragon* flower extracts and garlic-derived compounds have demonstrated multitarget binding affecting enzymes such as CYP51 and proteins involved in biofilm formation (Saqallah et al., 2022). A consistent observation across these studies is the pharmacological nature of plant-derived compounds. Unlike several synthetic antimicrobial drugs that act on a single molecular target, phytochemicals often exhibit moderate affinity for multiple microbial proteins, which may contribute to their broad-spectrum or synergistic antimicrobial activity.

Overall, recent comparative studies on plant extracts highlight the significance of *in silico* methods in antimicrobial research. These approaches reliably identify bioactive plant compounds capable of binding key microbial targets and prioritize candidates with the most favorable efficacy-to-toxicity profiles for further investigation (Ben Hassena et al., 2024; Dai et al., 2022). Although these *in silico* predictions provide valuable insights, experimental *in vitro* and *in vivo* validations are essential. For instance, a recent phytochemical screening study identified several plant-derived compounds as drug-like and emphasized extensive *in vitro* and *in vivo* validation to confirm their clinical relevance (Belitibo et al., 2024). This observation is consistent with our findings, demonstrating that computational predictions require empirical validation to ensure their reliability and biological significance.

## 5 Conclusion

This study highlights the significant antimicrobial properties of *O. europaea* and *F. carica* leaf extracts against MDR pathogens. Remarkably, *O. europaea* extracts, especially those derived using methanol, demonstrated potent antimicrobial activity against several pathogens. These findings are consistent with existing research, confirming that plant-derived compounds commonly exhibit antimicrobial properties by targeting microbial proteins. Nevertheless, this study identified certain limitations. *F. carica* extracts exhibited relatively narrow antimicrobial efficacy, aligning with previous evidence that factors such as extraction methodology, solvent system, the concentration of bioactive constituents, and overall phytochemical profile critically influence the therapeutic potential of plant-derived extracts. Although *O. europaea* extracts demonstrated encouraging therapeutic promise, *in vivo* studies revealed subtle signs of toxicity at higher doses, such as liver and kidney alterations, underscoring the importance of careful dose optimization. Although computational models predicted low risks for organ toxicity, notable discrepancies emerged when compared with experimental biological outcomes. This divergence between computational predictions and experimental findings underscores the limitation of *in silico* approaches. Although such models are valuable for early toxicity screening and microbial target identification, their utility must be integrated with *in vivo* and *in vitro* experiments. In conclusion, this study provided evidence supporting the antimicrobial potential of plant-derived phytochemicals against MDR pathogens while emphasizing the importance of therapeutic efficacy with rigorous safety evaluation. Further investigations are essential to establish optimal dosing strategies and to elucidate the molecular mechanisms underlying their biological activity before translation into clinical practice.

## Data Availability

The datasets presented in this study can be found in online repositories. The names of the repository/repositories and accession number(s) can be found in the article/Supplementary material.
