# Supplementary material for: Overall in vitro, in vivo, and in silico evaluation of Olea europaea and Ficus carica leaf extracts for antimicrobial activity against multidrug-resistant pathogens
Source: Front Microbiol. 2025 May 19;16:1567921. doi: 10.3389/fmicb.2025.1567921 (PMC12127348; doi:10.3389/fmicb.2025.1567921)

Supplementary Material

**Supplementary Table 1**. Antimicrobial activity of acetone extracts of *Olea europaea* and *Ficus carica* against selected clinical isolates, as determined by the well diffusion assay. Data represent the mean ± standard deviation (mm) of inhibition zone diameters obtained from three independent experiments. Each extract was tested at five concentrations: 100, 50, 25, 12.5, and 6.25 mg/mL. Levofloxacin (5 μg) and Sulconazole (25 μg) were included as positive controls for antibacterial and antifungal activity, respectively, while DMSO and acetone served as negative (solvent) controls.

| Bacterial /Fungal Isolates | Concentration of *O. europea* Extracts (mg/ml) | | | | | Concentration of *F. Carica* Extracts (mg/ml) | | | | | Positive Control | Negative Control (DMSO) | Negative Control  (Acetone) |
| --- | --- | --- | --- | --- | --- | --- | --- | --- | --- | --- | --- | --- | --- |
|  | 100 | 50 | 25 | 12.5 | 6.25 | 100 | 50 | 25 | 12.5 | 6.25 |  |  |  |
|  | Diameter of Inhibition Zone (mm) | | | | | | | | | | | |  |
| *E. faecium* | 6.00 ± 0.00 | 6.00 ± 0.00 | 6.00 ± 0.00 | 6.00 ± 0.00 | 6.00 ± 0.00 | 6.00 ± 0.00 | 6.00 ± 0.00 | 6.00 ± 0.00 | 6.00 ± 0.00 | 6.00 ± 0.00 | 18.00 ± 1.17 | 6.00 ± 0.00 | 6.00 ± 0.00 |
| *S. aureus* | 11.00 ± 0.00 | 6.00 ± 0.00 | 6.00 ± 0.00 | 6.00 ± 0.00 | 6.00 ± 0.00 | 6.00 ± 0.00 | 6.00 ± 0.00 | 6.00 ± 0.00 | 6.00 ± 0.00 | 6.00 ± 0.00 | 30.00 ± 1.00 | 6.00 ± 0.00 | 6.00 ± 0.00 |
| *S. agalactiae* | 10.00 ± 0.00 | 6.00 ± 0.00 | 6.00 ± 0.00 | 6.00 ± 0.00 | 6.00 ± 0.00 | 6.00 ± 0.00 | 6.00 ± 0.00 | 6.00 ± 0.00 | 6.00 ± 0.00 | 6.00 ± 0.00 | 20.33 ± 1.52 | 6.00 ± 0.00 | 6.00 ± 0.00 |
| *K. pneumoniae* | 14.67 ± 0.58 | 6.00 ± 0.00 | 6.00 ± 0.00 | 6.00 ± 0.00 | 6.00 ± 0.00 | 6.00 ± 0.00 | 6.00 ± 0.00 | 6.00 ± 0.00 | 6.00 ± 0.00 | 6.00 ± 0.00 | 30.33 ± 1.00 | 6.00 ± 0.00 | 6.00 ± 0.00 |
| *E. coli* | 14.33 ± 0.58 | 6.00 ± 0.00 | 6.00 ± 0.00 | 6.00 ± 0.00 | 6.00 ± 0.00 | 6.00 ± 0.00 | 6.00 ± 0.00 | 6.00 ± 0.00 | 6.00 ± 0.00 | 6.00 ± 0.00 | 16.67 ± 1.12 | 6.00 ± 0.00 | 6.00 ± 0.00 |
| *E. faecalis* | 6.00 ± 0.00 | 6.00 ± 0.00 | 6.00 ± 0.00 | 6.00 ± 0.00 | 6.00 ± 0.00 | 6.00 ± 0.00 | 6.00 ± 0.00 | 6.00 ± 0.00 | 6.00 ± 0.00 | 6.00 ± 0.00 | 10.67 ± 0.59 | 6.00 ± 0.00 | 6.00 ± 0.00 |
| *P. aeruginosa* | 6.00 ± 0.00 | 6.00 ± 0.00 | 6.00 ± 0.00 | 6.00 ± 0.00 | 6.00 ± 0.00 | 6.00 ± 0.00 | 6.00 ± 0.00 | 6.00 ± 0.00 | 6.00 ± 0.00 | 6.00 ± 0.00 | 24.33 ± 1.00 | 6.00 ± 0.00 | 6.00 ± 0.00 |
| *E. cloacae* | 6.00 ± 0.00 | 6.00 ± 0.00 | 6.00 ± 0.00 | 6.00 ± 0.00 | 6.00 ± 0.0 | 6.00 ± 0.00 | 6.00 ± 0.00 | 6.00 ± 0.00 | 6.00 ± 0.00 | 6.00 ± 0.00 | 21.00 ± 1.00 | 6.00 ± 0.00 | 6.00 ± 0.00 |
| *C. albicans* | 6.00 ± 0.00 | 6.00 ± 0.00 | 6.00 ± 0.00 | 6.00 ± 0.00 | 6.00 ± 0.00 | 6.00 ± 0.00 | 6.00 ± 0.00 | 6.00 ± 0.00 | 6.00 ± 0.00 | 6.00 ± 0.00 | 6.00 ± 0.00 | 6.00 ± 0.00 | 6.00 ± 0.00 |

**Supplementary Table 2**. Antimicrobial activity of methanol extracts of *Olea europaea* and *Ficus carica* against selected clinical isolates, as determined by the well diffusion assay. Data represent the mean ± standard deviation (mm) of inhibition zone diameters obtained from three independent experiments. Each extract was tested at five concentrations: 100, 50, 25, 12.5, and 6.25 mg/mL. Levofloxacin (5 μg) and Sulconazole (25 μg) were included as positive controls for antibacterial and antifungal activity, respectively, while DMSO and acetone served as negative (solvent) controls.

| Bacterial /Fungal Isolates | Concentration of *O. europea* Extracts ((mg/ml)) | | | | | Concentration of *F. Carica* Extracts ((mg/ml)) | | | | | Positive Control | Negative Control (DMSO) | Negative Control (Methanol) |
| --- | --- | --- | --- | --- | --- | --- | --- | --- | --- | --- | --- | --- | --- |
|  | 100 | 50 | 25 | 12.5 | 6.25 | 100 | 50 | 25 | 12.5 | 6.25 |  |  |  |
|  | Diameter of Inhibition Zone (mm) | | | | | | | | | | | |  |
| *E. faecium* | 28.34 ± 2.52 | 21.67 ± 2.08 | 6.00 ± 0.00 | 6.00 ± 0.00 | 15.33 ± 1.15 | 8.66 ± 0.57 | 7.67 ± 0.57 | 6.66 ± 0.57 | 6.67 ± 0.57 | 15.34 ± 1.15 | 15.33 ± 0.79 | 6.00 ± 0.00 | 6.00 ± 0.00 |
| *S. aureus* | 22.334 ± 14.15 | 19.34 ± 1.72 | 16.00 ± 10.00 | 12.00 ± 6.00 | 29.33 ± 4.16 | 20.00 ± 1.73 | 15.33 ± 1.15 | 9.66 ± 0.57 | 7.00 ± 0.00 | 29.334 ± 4.160 | 30.00 ± 1.12 | 6.00 ± 0.00 | 6.00 ± 0.00 |
| *S. agalactiae* | 29.00 ± 1.73 | 20.67 ± 1.70 | 14.67 ± 6.08 | 18.00 ± 0.00 | 20.33 ± 12.50 | 13.67 ± 2.08 | 10.66 ± 0.57 | 8.66 ± 0.57 | 6.00 ± 0.00 | 20.33 ± 12.50 | 6.00 ± 0.00 | 6.00 ± 0.00 | 6.00 ± 0.00 |
| *K. pneumoniae* | 25.67 ± 4.51 | 20.33 ± 4.04 | 13.33 ± 6.43 | 6.00 ± 0.00 | 34.33 ± 2.89 | 8.00 ± 3.46 | 8.00 ± 0.00 | 7.00 ± 0.00 | 6.00 ± 0.00 | 34.33 ± 2.89 | 27.50 ± 0.89 | 6.00 ± 0.00 | 6.00 ± 0.00 |
| *E. coli* | 42.66 ± 15.28 | 28.70± 1.70 | 19.00 ± 12.12 | 8.66 ± 4.62 | 6.00 ± 0.00 | 9.33 ± 5.77 | 6.00± 0.00 | 6.00 ± 0.00 | 6.00 ± 0.00 | 6.00 ± 0.00 | 34.33 ± 0.58 | 6.00 ± 0.00 | 6.00 ± 0.00 |
| *E. faecalis* | 21.33 ± 1.15 | 16.00 ± 0.00 | 9.00 ± 0.00 | 6.66 ± 0.57 | 24.00 ± 3.46 | 14.00± 0.00 | 6.00± 0.00 | 6.00 ± 0.00 | 6.00 ± 0.00 | 24.00 ± 3.46 | 23.00 ± 1.12 | 6.00 ± 0.00 | 6.00 ± 0.00 |
| *P. aeruginosa* | 22.67 ± 7.570 | 18.00 ± 1.58 | 12.67 ± 7.02 | 9.67 ± 6.35 | 6.00 ± 0.00 | 6.00± 0.00 | 6.00 ± 0.00 | 6.00 ± 0.00 | 6.00 ± 0.00 | 6.00 ± 0.00 | 6.00 ± 0.00 | 6.00 ± 0.00 | 6.00 ± 0.00 |
| *E. cloacae* | 39.33 ± 2.89 | 32.67 ± 2.89 | 26.00 ± 0.00 | 20.33± 5.15 | 29.33 ± 0.77 | 12.67 ± 2.89 | 6.00 ± 0.00 | 6.00 ± 0.00 | 6.00 ± 0.00 | 29.33 ± 5.77 | 31.00 ± 0.89 | 6.00 ± 0.00 | 6.00 ± 0.00 |
| *C. albicans* | 19.66 ± 1.52 | 11.66 ± 0.57 | 8.66 ± 1.15 | 19.66± 1.52 | 11.00± 0.66 | 6.00± 0.00 | 6.00 ± 0.00 | 6.00 ± 0.00 | 6.00 ± 0.00 | 11.00 ± 8.66 | 6.00 ± 0.00 | 6.00 ± 0.00 | 6.00 ± 0.00 |

**Supplementary Table 3**. Antimicrobial activity of aqueous extracts of *Olea europaea* and *Ficus carica* against selected clinical isolates, as determined by the well diffusion assay. Data represent the mean ± standard deviation (mm) of inhibition zone diameters obtained from three independent experiments. Each extract was tested at five concentrations: 100, 50, 25, 12.5, and 6.25 mg/mL. Levofloxacin (5 μg) and Sulconazole (25 μg) were included as positive controls for antibacterial and antifungal activity, respectively, while DMSO and acetone served as negative (solvent) controls.

| Bacterial /Fungal Isolates | Concentration of *O. europea* Extracts (mg/ml) | | | | | Concentration of *F. Carica* Extracts (mg/ml) | | | | | Positive Control | Negative Control (DMSO) | Negative Control (Water) |
| --- | --- | --- | --- | --- | --- | --- | --- | --- | --- | --- | --- | --- | --- |
|  | 100 | 50 | 25 | 12.5 | 6.25 | 100 | 50 | 25 | 12.5 | 6.25 |  |  |  |
|  | Diameter of Inhibition Zone (mm) | | | | | | | | | | | |  |
| *E. faecium* | 17.70 ± 0.79 | 6.00 ± 0.00 | 6.00 ± 0.00 | 6.00 ± 0.00 | 6.00 ± 0.00 | 6.00 ± 0.00 | 6.00 ± 0.00 | 6.00 ± 0.00 | 6.00 ± 0.00 | 6.00 ± 0.00 | 18.00 ± 1.17 | 6.00 ± 0.00 | 6.00 ± 0.00 |
| *S. aureus* | 20.3 ± 1.00 | 12.7 ± 0.58 | 7.3 0.43 | 6.00 ± 0.00 | 6.00 ± 0.00 | 11.33± 0.57 | 7.3 ± 0.43 | 6.00 ± 0.00 | 6.00 ± 0.00 | 6.00 ± 0.00 | 26.70 ± 0.89 | 6.00 ± 0.00 | 6.00 ± 0.00 |
| *S. agalactiae* | 6.00 ± 0.00 | 6.00 ± 0.00 | 6.00 ± 0.00 | 6.00 ± 0.00 | 6.00 ± 0.00 | 6.00 ± 0.00 | 6.00 ± 0.00 | 6.00 ± 0.00 | 6.00 ± 0.00 | 6.00 ± 0.00 | 6.00 ± 0.00 | 6.00 ± 0.00 | 6.00 ± 0.00 |
| *K. pneumoniae* | 14.70 ± 1.22 | 6.00 ± 0.00 | 6.00 ± 0.00 | 6.00 ± 0.00 | 6.00 ± 0.00 | 6.00 ± 0.00 | 6.00 ± 0.00 | 6.00 ± 0.00 | 6.00 ± 0.00 | 6.00 ± 0.00 | 20.30 ± 1.00 | 6.00 ± 0.00 | 6.00 ± 0.00 |
| *E. coli* | 14.70 ± 1.22 | 14.30 ± 1.22 | 6.00 ± 0.00 | 6.00 ± 0.00 | 6.00 ± 0.00 | 6.00 ± 0.00 | 6.00 ± 0.00 | 6.00 ± 0.00 | 6.00 ± 0.00 | 6.00 ± 0.00 | 24.00 ± 1.13 | 6.00 ± 0.00 | 6.00 ± 0.00 |
| *E. faecalis* | 6.00 ± 0.00 | 6.00 ± 0.00 | 6.00 ± 0.00 | 6.00 ± 0.00 | 6.00 ± 0.00 | 6.00 ± 0.00 | 6.00 ± 0.00 | 6.00 ± 0.00 | 6.00 ± 0.00 | 6.00 ± 0.00 | 16.73 ± 0.89 | 6.00 ± 0.00 | 6.00 ± 0.00 |
| *P. aeruginosa* | 27.00 ± 1.00 | 24.10 ± 1.12 | 16.00 ± 1.00 | 6.00 ± 0.00 | 6.00 ± 0.00 | 6.00 ± 0.00 | 6.00 ± 0.00 | 6.00 ± 0.00 | 6.00 ± 0.00 | 6.00 ± 0.00 | 6.00 ± 0.00 | 6.00 ± 0.00 | 6.00 ± 0.00 |
| *E. cloacae* | 23.10 ± 1.23 | 12.7 ± 1.43 | 6.00 ± 0.00 | 6.00 ± 0.00 | 6.00 ± 0.00 | 6.00 ± 0.00 | 6.00 ± 0.00 | 6.00 ± 0.00 | 6.00 ± 0.00 | 6.00 ± 0.00 | 24.32 ± 1.18 | 6.00 ± 0.00 | 6.00 ± 0.00 |
| *C. albicans* | 6.00 ± 0.00 | 6.00 ± 0.00 | 6.00 ± 0.00 | 6.00 ± 0.00 | 6.00 ± 0.00 | 6.00 ± 0.00 | 6.00 ± 0.00 | 6.00 ± 0.00 | 6.00 ± 0.00 | 6.00 ± 0.00 | 6.00 ± 0.00 | 6.00 ± 0.00 | 6.00 ± 0.00 |

**Supplementary Table 4.** *In vitro* profiling of organ-specific toxicities and key toxicological endpoints associated with olive leaf extract. The “Classification” column categorizes the endpoint as either organ toxicity or a broader toxicological mechanism. The “Target” column specifies the organ system or molecular endpoint assessed. The “Prediction” column indicates whether the extract induced a measurable response, classified as ‘Active’ (suggesting a potential adverse effect) or ‘Inactive’ (no significant response). The “Probability” column reflects the assay’s confidence in the observed signal, with values approaching 1.00 indicating high reliability.

| **Classification** | **Target** | **Prediction** | **Probability** |
| --- | --- | --- | --- |
| Organ toxicity | Hepatotoxicity | Inactive | 0.85 |
| Organ toxicity | Neurotoxicity | Inactive | 0.88 |
| Organ toxicity | Nephrotoxicity | **Active** | 0.75 |
| Organ toxicity | Respiratory toxicity | Inactive | 0.54 |
| Organ toxicity | Cardiotoxicity | **Active** | 0.77 |
| Toxicity endpoint | Carcinogenicity | Inactive | 0.79 |
| Toxicity endpoint | Immunotoxicity | **Active** | 0.98 |
| Toxicity endpoint | Mutagenicity | Inactive | 0.84 |
| Toxicity endpoint | Cytotoxicity | Inactive | 0.70 |
| Toxicity endpoint | BB-barrier | **Active** | 0.52 |
| Toxicity endpoint | Ecotoxicity | Inactive | 0.70 |
| Toxicity endpoint | Clinical toxicity | **Active** | 0.64 |
| Toxicity endpoint | Nutritional toxicity | **Active** | 0.54 |

**Supplementary Table 5.** *In vitro* evaluation of olive leaf extract activity on nuclear receptor signaling pathways using the ToxProfile™ platform. The “Classification” column denotes the pathway category, and the “Target” column identifies the specific receptor or ligand-binding domain assessed. The “Prediction” column indicates whether the extract elicited a measurable biological response classified as 'Active' (indicating potential interaction) or 'Inactive' (no significant interaction). The “Probability” column reflects the confidence in each response, with values closer to 1.00 denoting greater assay reliability.

| **Classification** | **Target** | **Prediction** | **Probability** |
| --- | --- | --- | --- |
| Tox21 nuclear receptor signaling pathway | Aryl hydrocarbon receptor (AHR) | Inactive | 0.94 |
| Tox21 nuclear receptor signaling pathway | Androgen receptor (AR) | Inactive | 0.97 |
| Tox21 nuclear receptor signaling pathway | Androgen receptor ligand binding domain (AR-LBD) | Inactive | 0.97 |
| Tox21 nuclear receptor signaling pathway | Aromatase | Inactive | 0.86 |
| Tox21 nuclear receptor signaling pathway | Estrogen receptor alpha | Inactive | 0.75 |
| Tox21 nuclear receptor signaling pathway | Estrogen receptor ligand binding domain (LBD) | Inactive | 0.97 |
| Tox21 nuclear receptor signaling pathway | Peroxisome proliferator-activated receptor gamma(PPAR-Gamma) | Inactive | 0.92 |

**Supplementary Table 6.** *In vitro* assessment of olive leaf extract activity on key cellular stress response pathways using the ToxProfile™ platform. The “Classification” column denotes the specific type of stress response pathway, while the “Target” column identifies the transcriptional reporter associated with each stress-related factor. The “Prediction” column indicates whether the extract induced a measurable transcriptional response classified as 'Active' or 'Inactive.' The “Probability” column provides the confidence level of each response, with values closer to 1.00 representing higher assay reproducibility and biological significance

| **Classification** | **Target** | **Prediction** | **Probability** |
| --- | --- | --- | --- |
| Tox21 Stress response pathways | Nuclear factor (erythroid-derived 2)-like 2/antioxidant responsive element (nrf2/ARE) | Inactive | 0.92 |
| Tox21 Stress response pathways | Heat shock factor response element (HSE) | Inactive | 0.92 |
| Tox21 Stress response pathways | Mitochondrial Membrane Potential (MMP) | Inactive | 0.82 |
| Tox21 Stress response pathways | Phosphoprotein (Tumor Suppressor) p53 | Inactive | 0.79 |
| Tox21 Stress response pathways | ATPase family AAA domain-containing protein 5 (ATAD5) | Inactive | 0.97 |

**Supplementary Table 7**. *In vitro* profiling of olive leaf extract interactions with molecular initiating events (MIEs) using the ToxProfile™ platform. The “Classification” column indicates the category of the initiating event, while the “Target” column lists the specific receptor, enzyme, or molecular mechanism evaluated. The “Prediction” column denotes whether the extract elicited a transcriptional response ('Active') or not ('Inactive'). The “Probability” column reflects the confidence level of the measured activity, with values approaching 1.00, indicating stronger assay reproducibility and biological significance.

| **Classification** | **Target** | **Prediction** | **Probability** |
| --- | --- | --- | --- |
| Molecular Initiating Events | Thyroid hormone receptor alpha (THRα) | Inactive | 0.90 |
| Molecular Initiating Events | Thyroid hormone receptor beta (THRβ) | Inactive | 0.78 |
| Molecular Initiating Events | Transtyretrin (TTR) | Inactive | 0.97 |
| Molecular Initiating Events | Ryanodine receptor (RYR) | Inactive | 0.98 |
| Molecular Initiating Events | GABA receptor (GABAR) | Inactive | 0.96 |
| Molecular Initiating Events | Glutamate N-methyl-D-aspartate receptor (NMDAR) | Inactive | 0.92 |
| Molecular Initiating Events | alpha-amino-3-hydroxy-5-methyl-4-isoxazole-propionate receptor (AMPAR) | Inactive | 0.97 |
| Molecular Initiating Events | Kainate receptor (KAR), Acetylcholinesterase (AChE) | Inactive | 0.99 |
| Molecular Initiating Events | Constitutive androstane receptor (CAR) | Inactive | 0.85 |
| Molecular Initiating Events | Pregnane X receptor (PXR) | Inactive | 0.98 |
| Molecular Initiating Events | NADH-quinone oxidoreductase (NADHOX) | Inactive | 0.92 |
| Molecular Initiating Events | Voltage-gated sodium channel (VGSC) | Inactive | 0.97 |
| Molecular Initiating Events | Na+/I- symporter (NIS) | Inactive | 0.95 |


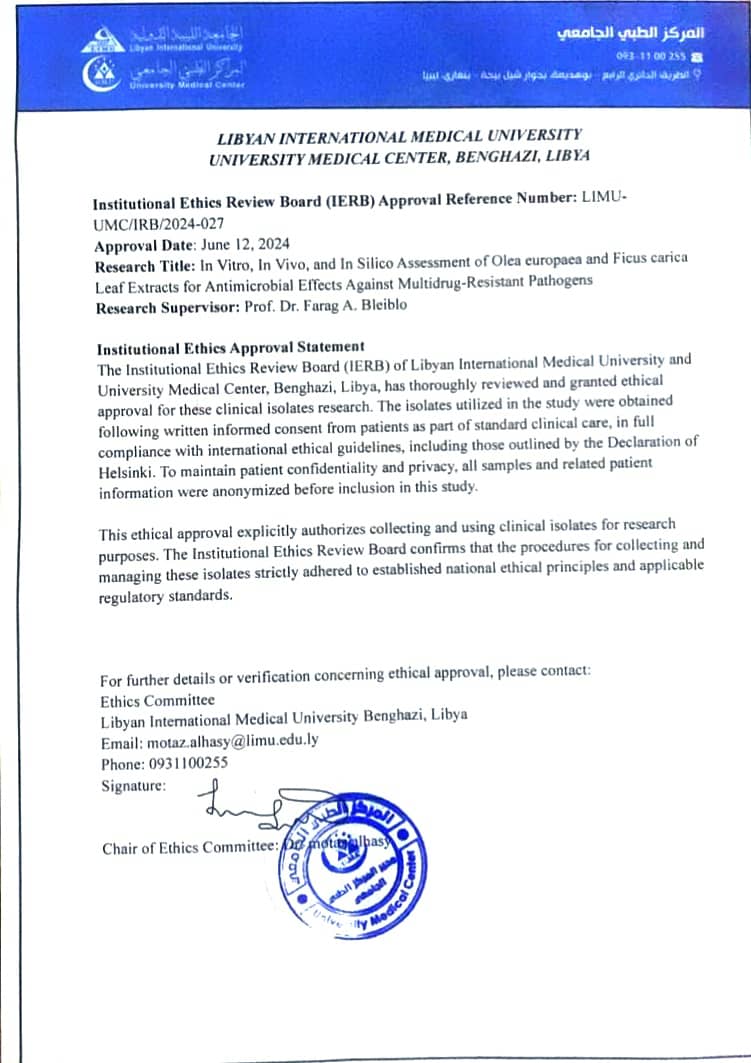


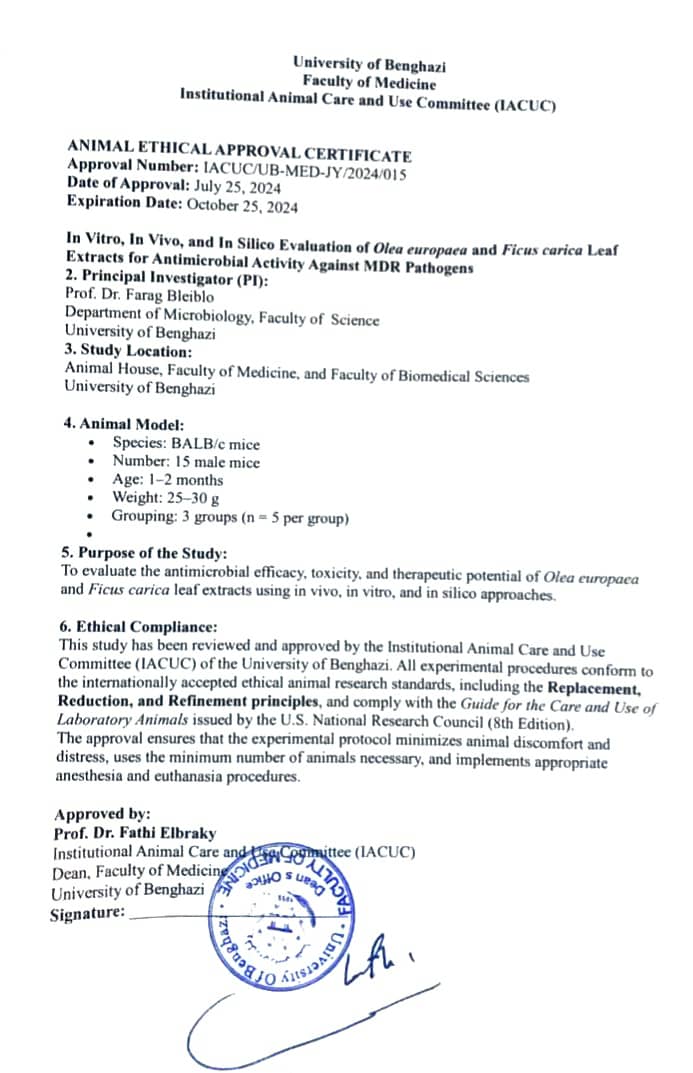

Supplement: Supplementary file 1 [file Data_Sheet_1.docx]
